# Contraceptive effectiveness, pharmacokinetics, and safety of Sayana® Press when injected every four months: a multicenter phase 3 trial

**DOI:** 10.1016/j.eclinm.2022.101273

**Published:** 2022-01-29

**Authors:** Jennifer Deese, Vivian Brache, Luis Bahamondes, Abril Salinas, Aidelis Jorge, Nelio Veiga, Rachael Fuchs, Ashley Miller, Doug Taylor, Vera Halpern, Laneta Dorflinger

**Affiliations:** a Women's Global Health Imperative, Global Public Health Impact Center, RTI International, 3040 East Cornwalis Road, Research Triangle Park, NC 27709, United States; b Profamilia, Santo Domingo, Dominican Republic; c Department of Obstetrics and Gynaecology, University of Campinas Faculty of Medical Sciences, Campinas, Sao Paulo, Brazil; d Instituto Chileno de Medicina Reproductiva, Universidad de Chile, Chile; e Biostatistics and Data Sciences, FHI 360, 359 Blackwell Street, Durham, NC 27701, United States; f Science Facilitation, FHI 360, 359 Blackwell Street, Durham, NC 27701, United States; g Product Development and Introduction, FHI 360, 359 Blackwell Street, Durham, NC 27701, United States

**Keywords:** Depot medroxyprogesterone acetate, Contraception, Contraceptive effectiveness, Contraceptive pharmacokinetics, Subcutaneous

## Abstract

**Background:**

Sayana Press® is a 3-monthly contraceptive injection approved by regulatory agencies in more than 40 countries worldwide. Existing effectiveness and pharmacokinetics (PK) data suggest that high contraceptive efficacy may be maintained if the reinjection interval of Sayana Press is extended from 3 to 4 months.

**Methods:**

We conducted a phase 3 trial at three sites in the Dominican Republic, Brazil, and Chile from September 2017 through April 2020. We enrolled 750 women at risk of pregnancy who agreed to use Sayana Press off-label every 4 months (3 treatment cycles) for 12 months. The effectiveness cohort included 710 participants randomized equally to receive injections in the abdomen or thigh. Forty additional participants received injections in the back of the upper arm for comparative PK analyses. The primary outcome was pregnancy, defined by a positive urine pregnancy test confirmed by ultrasound and/or serum human chorionic gonadotropin. Secondary outcomes included PK, safety, and acceptability. Laboratory and trial Sponsor staff were blind to injection site. This study is registered with ClinicalTrials.gov, number NCT03154125.

**Findings:**

There were no pregnancies during follow-up; the Pearl Index during 629.3 woman-years (WY) of follow-up in the primary effectiveness analysis was 0.00 (95% CI 0.00, 0.59). Pharmacokinetic profiles differed by injection site, with higher geometric mean (GM) medroxyprogesterone acetate concentrations for the abdomen than the thigh and arm. At month 8, significantly higher GM concentrations were observed in the abdomen and the thigh as compared to the arm, as well as at month 12 in the abdomen as compared to the arm. Injection site reactions were reported by 10.7% of participants.

**Interpretation:**

Both pregnancy and PK results confirm that Sayana Press is a highly effective contraceptive method when administered every 4 months. These findings may inform modification of the dosing schedule, or duration of the grace period for reinjection, or both, to reduce overall drug exposure while maintaining contraceptive efficacy.

**Funding:**

This work is made possible by the generous support of the American people through the U.S. Agency for International Development (USAID), provided to FHI 360 through Cooperative Agreement AID-OAA-A-15–00,045, and a grant from the Gates Foundation. The contents are the responsibility of FHI 360 and do not necessarily reflect the views of USAID, the United States Government, or the Gates Foundation, nor does any mention of trade names, commercial products, or organizations imply endorsement by FHI 360, USAID, the United States Government, or the Gates Foundation.

**Research in Context**

**Evidence before this study**

Sayana Press is labeled for injection at three-month intervals. We conducted a meta-analysis of publicly available pharmacokinetic and pharmacodynamic data (including a study that informed the Depo-subQ label, a study which assessed pharmacokinetics of different injection sites, and a public assessment report for Sayana® Press) for Depo-subQ Provera 104 to evaluate whether contraceptive effectiveness may be maintained when extending the injection interval to four months.

**Added value of this study**

We performed a randomized, multicenter study to assess the efficacy of Sayana Press when injected every 4 months in the abdomen or thigh in accordance with the European Medicines Agency Guideline on Clinical Investigation of Steroid Contraceptives in Women. Results confirmed a high degree of efficacy for the 4-month regimen in a diverse population of women.

**Implications of all the available evidence**

The reinjection interval for Sayana Press, and by extension for Depo-subQ Provera 104, can be increased from 3 to 4 months with little risk of pregnancy and less drug exposure. This has the potential to reduce user and health system costs and improve acceptability among women who prefer less frequent injections.

Alt-text: Unlabelled box

## Introduction

Globally, over 74 million women use injectable contraceptives for pregnancy prevention.^1^ Depot medroxyprogesterone acetate (DMPA) for intramuscular (IM) administration every three months (150 mg/mL medroxyprogesterone acetate [MPA] injectable suspension; DMPA-IM) is the most prevalent injectable method. Although widely used, DMPA-IM is associated with weight gain, bleeding disturbances, metabolic effects, reduced bone mineral density^2^, ^3^, ^4^, ^5^, ^6^, ^7^ and delayed return to fertility.^8^ More recently, Pfizer developed Depo-SubQ Provera 104®, an alternative MPA formulation (104 mg/0.65 mL) for subcutaneous (SC) administration every 3 months.

Due in part to a slower rate of absorption following SC administration, Depo-SubQ Provera 104 achieves the same degree of efficacy as DMPA-IM at a 31% lower dose.^9^^,^^10^ Despite the lower dose, steady-state trough MPA concentrations of the two drugs are similar. In a randomized study, average serum MPA concentrations 1 and 2 years after initiating Depo-SubQ Provera 104 were 100% and 85%, respectively of the corresponding DMPA-IM values.^10^ This, combined with the lack of any pregnancies in phase 3 trials of Depo-SubQ Provera 104^9^^,^^10^ indicates that the 104 mg dose may be higher than necessary for 3-month protection and that the duration of its action may be extended from 3 to 4 months. This would reduce long-term MPA exposure by 25% and lower steady state trough MPA concentrations, thus possibly shortening the delay in return to fertility and improving other dose-dependent side effects.

Because of the ease of SC administration, Sayana® Press (Depo-SubQ Provera 104 in the all-in-one prefilled, auto-disabled injection system called Uniject™), also manufactured by Pfizer and approved for self-injection, has the potential to increase contraceptive access in settings where resource constraints are a barrier to provision and consistent use of injectable contraception.^11^ Sayana Press is already approved in more than 40 countries and its ongoing scale-up is supported by large international family planning donors.^12^ A 4-month reinjection interval would decrease programmatic costs and further facilitate access to Sayana Press in resource-constrained settings.

We hypothesized that a high degree of contraceptive efficacy would be maintained when extending the reinjection interval of Sayana Press to 4 months. To test this hypothesis, we conducted a study to evaluate the effectiveness, safety, and acceptability of Sayana Press when injected every 4 months in the abdomen or thigh for 12 months of use (3 treatment cycles). In addition, we assessed the impact of injection site (abdomen, thigh, or arm), study center, body mass index, and age on pharmacokinetic profiles.

## Methods

### Study design and participants

We conducted a phase 3, randomized, non-comparative, multicenter, partially blinded trial at three research centers: Biomedical Research Department at PROFAMILIA (Santo Domingo, Dominican Republic); Family Planning Clinic, Department of Obstetrics and Gynaecology, Faculty of Medical Sciences, School of Medicine, University of Campinas (Campinas, São Paulo, Brazil), and Instituto Chileno de Medicina Reproductiva (ICMER) (Santiago, Chile). We enrolled women aged 18–35 years who were not pregnant or lactating and did not desire pregnancy for the next 18 months; had regular menstrual cycles (25 to 35 days in length when not using hormonal contraception, pregnant, or lactating); were at risk of pregnancy; had not received an injection of a combined injectable contraceptive in the prior six months or DMPA in the prior 12 months and were willing to rely solely on Sayana Press for contraception for the duration of their trial participation (see protocol in the appendix for complete eligibility criteria). Additionally, women who had used a hormonal intrauterine device, NuvaRing®, contraceptive patch or oral contraceptives in the 7 days prior to enrollment were not eligible for participation in the pharmacokinetics (PK) cohort.

Ethics review committees at each center and the FHI 360´s Human Protection Committee approved the study protocol and informed consent forms. All participants provided written informed consent prior to study participation and were reimbursed for their time and transportation costs for each study visit in accordance with local Institutional Review Board approvals. The trial was conducted in accordance with International Council for Harmonization Good Clinical Practice (GCP) guidelines.^13^ This study was registered with ClinicalTrials.gov (NCT03154125). The trial design and results are reported in this manuscript following the Consolidated Standards of Reporting Trials (CONSORT) guideline.

### Randomization and masking

We randomized 630 participants at a 1:1 allocation to receive Sayana Press injections in the abdomen or thigh and 120 participants for a pharmacokinetics (PK) cohort at a 1:1:1 allocation to receive injections in the abdomen, thigh, or arm, for a total of 750 participants. Randomization was stratified by study center using randomly permuted block sizes; randomization sequences were generated by an independent statistician not otherwise affiliated with the study. Randomized assignments were concealed in sealed, sequentially numbered, opaque envelopes accessible only to designated clinical staff responsible for participant randomization. Neither participants nor study staff at the research centers were blinded to injection site; however, laboratory and Sponsor staff (including the trial Principal Investigator, Medical Director, data staff and statisticians) were blinded to injection site.

### Procedures

All participants attended screening, enrollment, and follow-up visits at months 4, 8 and 12. The first injection of Sayana Press was administered at enrollment within the first 5 days of menses in accordance with standardized injection instructions.^14^ Infrequently during administration, Uniject devices became blocked and did not dispense the drug in which case staff tried to resolve the blockage by turning the needle without removing it, and used a new device when the blockage could not be resolved. Reinjections were scheduled at month 4 (day 119) and month 8 (day 245) following the first injection with an allowable +7-day grace period and administered at the randomized injection site. Participants who were between 8 and 28 days late for reinjections could continue in the study and receive their reinjection upon a confirmed negative urine pregnancy test; subsequent follow-up visits were rescheduled to ensure at least 126-day intervals between visits. Participants who were more than 28 days late for reinjections were discontinued. Urine pregnancy testing was done at enrollment, months 4 and 12, and at any other time if clinically indicated. Additionally, at months 4, 8 and 12, blood pressure and weight measurements were taken, and participants were evaluated for injection site reactions (ISRs), asked to provide information on adverse events (AE), prohibited concomitant medication and other contraceptive use, vaginal bleeding, coital frequency, and method acceptability. Month 12 visit procedures were done at the time of early discontinuation when possible.

In addition, participants in the PK cohort provided serum specimens for MPA testing at baseline, months 2, 3, 4, 8 and 12.

### Outcomes

The primary outcome was the occurrence of pregnancy after enrollment, defined by a positive urine pregnancy test confirmed by ultrasound and/or serum human chorionic gonadotropin testing. Secondary outcomes included PK parameters, safety, and acceptability. PK outcomes included MPA concentrations at months 2, 3, 4, 8, and 12, accumulation, and apparent terminal half-life of MPA estimated based on MPA concentrations at months 2, 3, and 4. We evaluated the impact of injection site, study center, body mass index (BMI; kg/m^2^), and age on PK profiles. Serum samples were prepared and immediately frozen at approximately −20 °C at the investigational sites and shipped on dry ice to PPD Development (Richmond, VA, USA) for analysis. PPD measured serum MPA concentrations using a validated high-performance liquid chromatography tandem mass spectrometry assay (inter-assay and intra-assay precision, expressed as the coefficient of variation times 100, ranged from 8.35% to 43.6% (10.7 excluding Dixon outlier) and from 10.8% to 90.3%, respectively). This method is applicable to quantitation within a nominal MPA range of 0.0200 to 5.00 ng/mL.

Given the well-established safety profile of Sayana Press^15^ and lower overall drug exposure over 12 months compared to the approved 3-month regimen, we limited safety reporting in this study to serious AEs (SAEs), AEs leading to product withdrawal, ISRs, blood pressure, and body weight at months 4, 8 and 12. Bleeding was assessed at scheduled follow-up visits or time of early discontinuation by questionnaire which included, but was not limited to, questions about average cycle length, duration of flow, amount of flow and a description of bleeding/spotting pattern since her last injection. Injection sites were observed by clinical staff at approximately 15 min following each injection, and participants were asked at subsequent visits if any reaction(s) occurred following their last injection. Severity of ISRs was graded using the Division of AIDS (DAIDS) Table or Grading the Severity of Adult and Pediatric Adverse Events^16^; ISRs of Grade 1 or higher were reported as AEs. Acceptability outcomes included responses to questions about perception of bleeding patterns (at enrollment, months 4, 8, and 12) and other side effects, likes, and dislikes about the regimen (recorded only at month 12 visit or early discontinuation), and will be more comprehensively reported in a separate manuscript.

### Statistical analysis

The effectiveness cohort sample size of 710 women receiving injections in the abdomen or thigh was designed to be sufficient to ensure that the difference between the estimated Pearl Index and the corresponding upper 95% confidence bound did not exceed 1.0, under the assumption that the observed index would be less than 0.75 and at least 80% of participants completed 12 months of follow-up, in accordance with the European Medicines Agency Guideline on Clinical Investigation of Steroid Contraceptives in Women.^17^ The primary effectiveness analysis included all enrolled participants who received at least one Sayana Press injection in the abdomen or thigh and included all follow-up time contributed by those participants until the earliest of their estimated date of fertilization, the end of their treatment period, or the date of their last negative urine pregnancy test (for participants who did not become pregnant). Follow-up time contributed by a woman when she was 8 to 28 days late for a reinjection was excluded from all effectiveness analyses. Supportive sub-group analyses were also conducted by randomized site of injection. The pregnancy Pearl Index was computed as the number of pregnancies that occurred during the treatment period multiplied by 100 and divided by the number of woman-years of treatment; exact 95% confidence intervals (CI) were computed based on a Poisson distribution assumption.

The PK cohort sample size of 120 women receiving injections in the abdomen, thigh or arm was designed to provide 85% power to detect 30% relative differences in PK parameters between injection sites, assuming the corresponding coefficient of variation is no more than 40% and using two-sided 0.05 level significance tests. PK analyses were conducted among the Pharmacokinetics Population, which excluded participants who did not contribute specimens for PK assessments or who had a baseline serum MPA concentration >0.05 ng/mL. PK analyses also excluded results from samples collected during concomitant medication use that might have impacted PK of MPA and samples collected after an injection which required the use of a second Sayana Press due to blockage of the first device. Geometric means (GMs), percent coefficients of variation, and upper and lower 10th percentiles of MPA concentrations were calculated by injection site. Geometric mean ratios (GMR) and 95% CI based on log-transformed responses were used to compare trough concentrations across injection sites. Mixed-effects log-linear models were used to assess rates of MPA decrease in the two months prior to reinjection at month 4 (a surrogate for apparent half-life), trough accumulation ratio (ARs) at month 12 (predicted GM MPA at month 12 versus month 4), and covariate effects on trough MPA concentrations.

Safety analyses included all follow-up time contributed by each woman. Numbers and percentages of participants experiencing reportable AEs (i.e., SAEs, AEs leading to product withdrawal and ISRs which were Grade 1 or higher) were compared across injection sites within system organ class using Fisher's exact tests. Numbers and percentages of participants experiencing ISRs, irrespective of grade, were calculated by injection site.

The DSMB conducted an interim review when 250 participants had completed 4 months of follow-up to inform a decision to stop or modify the study if the estimated probability of pregnancy was greater than 2% in the first injection interval. Analyses were generated using SAS/STAT software, Version 9.4 of the SAS System for Windows.

### Role of the funding source

The study funders were not involved in the design or execution of the trial, interpretation of results, or decision to submit for publication. The authors designed and implemented the study, performed all analyses, affirm data completeness, wrote the manuscript, and were responsible for the decision to submit for publication. All authors had access to the study data.

## Results

### Study participants and follow-up

**Figure 1:**
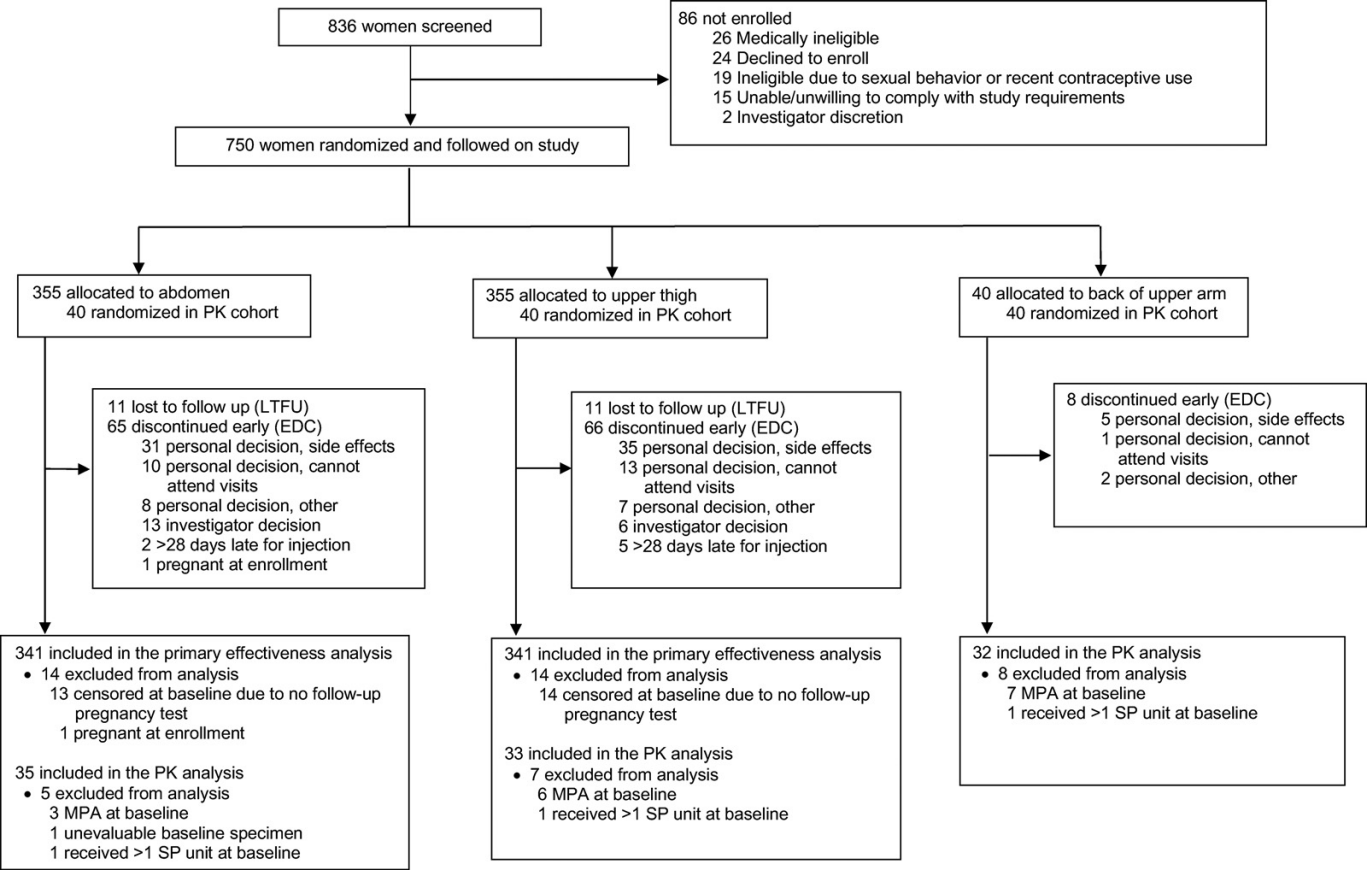
Trial Profile. PK = pharmacokinetics.

Between September 2017 and April 2019, we enrolled and randomized a total of 750 women as follows: 355 to receive injections in the abdomen, 355 in the thigh and 40 in the arm (Figure 1); follow-up of participants was completed in March 2020. As planned, 710 women were included in the effectiveness cohort (all participants randomized to receive injections in the abdomen or thigh) and 120 women were included in the PK cohort (40 per each of the three injection sites); the 80 women randomized to receive injections in the abdomen or thigh in the PK cohort were also included in the effectiveness cohort. There were no randomization or treatment allocation errors. The median age of enrolled women was 25 (IQR 21 to 29) (Table 1). The racial composition of the study included 228 (30.4%) women who identified as white, 58 (7.7%) as black and 464 (61.9%) as biracial. Four hundred thirty-one (57.5%) were married or living with their partner, and 319 (42.5%) had a regular non-cohabitating partner. Participants reported a median duration of menstrual bleeding of 5.0 days (IQR 4 to 6), a median of 1.0 (IQR 0 to 2) prior pregnancies and a median of 10.0 (IQR 6 to 15) vaginal sex acts per month with their partner. Baseline characteristics were well balanced by randomized injection site in both the effectiveness and PK cohorts.

**Table 1:** Baseline characteristics of study participants.

	Abdomen (*n* = 355)	Upper Thigh (*n* = 355)	Back of Upper Arm (*n* = 40)	Overall (*n* = 750)
Age (years)	25 (21 to 30)	25 (21 to 29)	24 (23 to 28)	25 (21 to 29)
Partner Status				
married/cohabitating	211 (59%)	202 (57%)	18 (45%)	431 (57%)
regular non-cohabitating partner	144 (41%)	153 (43%)	22 (55%)	319 (43%)
Race				
white	104 (29%)	104 (29%)	20 (50%)	228 (30%)
black	28 (8%)	29 (8%)	1 (3%)	58 (8%)
bi-/multi-racial	223 (63%)	222 (63%)	19 (48%)	464 (62%)
Body mass index, (kg/m^2^)	27 (23 to 33)	27 (23 to 31)	28 (24 to 32)	27 (23 to 32)
Duration of menstrual bleeding (days)	5 (4 to 5)	5 (4 to 6)	5 (4 to 6)	5 (4 to 6)
Any prior pregnancy	271 (76%)	254 (72%)	26 (65%)	551 (73%)
Vaginal sex acts per month	12 (6 to 16)	10 (6 to 15)	10 (6 to 12)	10 (6 to 15)
Never use condoms for STI/HIV prevention	141 (40%)	157 (44%)	25 (63%)	323 (43%)
Contraceptive history^1^				
DMPA or NET-EN	157 (44%)	135 (38%)	12 (30%)	304 (41%)
Combined injectable	43 (12%)	66 (19%)	4 (10%)	113 (15%)
LNG-IUS	4 (1%)	4 (1%)	0 (0%)	8 (1%)
NuvaRing	7 (2%)	12 (3%)	2 (5%)	21 (3%)
Patch	7 (2%)	1 (<1%)	0 (0%)	8 (1%)
Pills	220 (62%)	218 (61%)	31 (78%)	469 (63%)
Implant	49 (14%)	61 (17%)	6 (15%)	116 (15%)
Condoms	250 (70%)	243 (68%)	25 (63%)	518 (69%)
Other	71 (20%)	62 (17%)	5 (13%)	138 (18%)
None	6 (2%)	7 (2%)	0 (0%)	13 (2%)

Data are n (%) or median (IQR). DMPA=depot medroxyprogesterone acetate; NET-EN=norethisterone enanthate; LNG IUS=levonorgestrel intrauterine system.

1 Multiple responses may apply.

A total of 589 (78.5%) participants completed the study, 139 (18.5%) discontinued early and 22 (2.9%) were lost to follow-up (Figure 1). Most early discontinuations (*n* = 112; 80.6%) were due to personal reasons including side effects (*n* = 71; 63.4%) and inability to attend study visits (*n* = 24; 21.4%), followed by investigator decision (*n* = 19; 13.7%), being more than 28 days late for a scheduled injection (*n* = 7; 5.0%) and one participant who was determined to have been pregnant at the enrollment visit. The demographic characteristics of participants who completed the study were similar to those of participants who discontinued early. Most (65.8%) follow-up reinjections were administered on the scheduled day, 29.9% in the 7-day grace period and 2.1% in the late 28-day period. Among the 120 women randomized to the PK cohort, 20 (16.7%) were excluded from PK analyses due to unevaluable baseline MPA specimen (*n* = 1), baseline MPA concentration >0.05 ng/mL (*n* = 16) or use of a second device due to Uniject malfunction (*n* = 3).

### Effectiveness

No pregnancies were observed during the follow-up treatment period. Twenty-eight of 710 (3.9%) women randomized to receive injections in the abdomen or thigh were excluded from the primary effectiveness analysis, including one participant determined to have become pregnant based on the estimated date of fertilization on the day of enrollment and 27 who discontinued early or were lost to follow-up prior to having any follow-up pregnancy test. The remaining 682 women contributed 629.3 woman-years (WY) of follow-up, resulting in a Pearl Index of 0.00 (95% CI 0.00, 0.59) (Table 2). Participants reported a median of 11 (IQR 7 to 16) vaginal sex acts per month during follow-up and the majority (78%) reported never having used condoms. A perfect use analysis was defined *a priori* among the effectiveness cohort which excluded any time during which a participant reported using another contraceptive method (including condoms; 36.2 WY excluded) or drugs known to impact ovarian function (0.3 WY excluded) or the PK of MPA (0.1 WY excluded). Therefore, the perfect use analysis included 592.7 WY of follow-up, resulting in a Pearl Index of 0.00 (95% CI 0.00, 0.62). Moreover, we conducted a sensitivity analysis which reduced person-time based on reported vaginal sex and condom use among the effectiveness cohort (Table 2), and the estimated proportion of participants in the PK cohort with >0.05 ng/mL MPA at baseline (14.0%). This sensitivity analysis included 500.5 WY of follow up, resulting in a Pearl Index of 0.00 (95% CI 0.00, 0.74).

**Table 2:** Contraceptive Effectiveness Measured by Pearl Index and 95% Confidence Interval among Participants Receiving Injections in the Abdomen and Upper Thigh.

	No. of Women	Person Years	No. of Pregnancies	Pearl Index (per 100 WY)	95% CI for Pearl Index
Primary Effectiveness^1^	682	629.3	0	0.00	(0.00, 0.59)
Perfect Use^2^	680	592.7	0	0.00	(0.00, 0.62)
Sensitivity Analysis^3^	682	500.5	0	0.00	(0.00, 0.74)

1 28 out of 710 women were censored at enrollment and are not included in this count. 10 were lost to follow-up, 17 discontinued early without a pregnancy test, and 1 was already pregnant at enrollment.

2 30 out of 710 women were censored from the Perfect Use analysis at enrollment and are not included here.

3 Pearl Index computed using a conservative scenario in terms of person-time. Approximately 14% of women in the PK cohort had baseline MPA >= 0.05 ng/mL. Also 81.4%, 84.2%, and 85.5% of women reported having sex in the previous 4 weeks (time most at risk) and having no condom use since last injection for baseline, month 4, and month 8 injection cycles respectively. Based on these estimates, we included 70% (86%*81.4%) of WY contributed in the first injection cycle, and 84.2% and 85.5% of WY contributed in the second and third injection cycles, respectively.

### Pharmacokinetics

**Figure 2:**
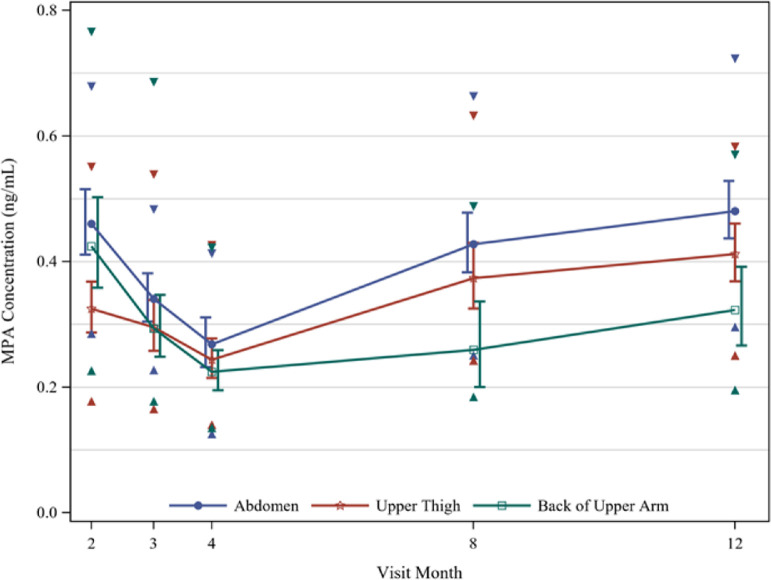
Geometric Mean MPA Concentrations by Injection Site with 90% CIs (shifted slightly for visibility) summarized among the PK Population. Triangles denote 10th and 90th Percentiles at each timepoint.

Month 4 GM trough concentrations were 21%, 20%, and 24% lower than trough concentrations at month 3 for the abdomen, thigh, and arm, respectively. At month 4, 18%, 29%, and 32% of women in these respective groups had MPA concentrations below 0.2 ng/mL and about 10% in all three groups had concentrations below 0.14 ng/mL (Table 3). Pharmacokinetic profiles differed by injection site, with higher GM concentrations for the abdomen (0.34, 0.27, 0.43, and 0.48 ng/mL at months 3, 4, 8, and 12, respectively) than the thigh (0.30, 0.24, 0.37, 0.41 ng/mL) and arm (0.29, 0.22, 0.26, 0.32 ng/mL) (Figure 2). At month 8, significantly higher GM concentrations were observed in the abdomen and the thigh as compared to the arm (GMR [95% CI]: 1.65 [1.17, 2.32] and 1.44 [1.01, 2.06] in the abdomen and thigh, respectively), as well as at month 12 in the abdomen as compared to the arm (GMR [95% CI]: 1.49 [1.15, 1.94]) (Table 3).

**Table 3:** MPA Concentrations (ng/mL) by Injection Site, PK Population.

				Geometric Mean Ratio (95% CI)		
	Abdomen	Upper Thigh	Back of Upper Arm	Abdomen vs. Thigh	Abdomen vs. Arm	Thigh vs. Arm
C_2mo_						
GM (%CV)	0.46 (52.4)	0.32 (43.7)	0.42 (54.0)	1.42 (1.15, 1.74)	1.08 (0.85, 1.39)	0.77 (0.59, 0.99)
P_10_ - P_90_*	0.29 - 0.68	0.18 - 0.55	0.23 - 0.77			
% < 0.2 ng/mL	0.0	12.5	6.5			
Total	34	32	31			
C_3mo_						
GM (%CV)	0.34 (39.5)	0.30 (48.9)	0.29 (53.3)	1.15 (0.93, 1.43)	1.16 (0.91, 1.49)	1.01 (0.77, 1.31)
P_10_ - P_90_*	0.23 - 0.48	0.17 - 0.54	0.18 - 0.69			
% < 0.2 ng/mL	8.8	22.6	16.1			
Total	34	31	31			
C_4mo_						
GM (%CV)	0.27 (43.7)	0.24 (42.3)	0.22 (42.3)	1.10 (0.86, 1.40)	1.20 (0.93, 1.54)	1.09 (0.86, 1.38)
P_10_ - P_90_*	0.13 - 0.41	0.14 - 0.43	0.14 - 0.42			
% < 0.2 ng/mL	18.2	29.0	32.3			
Total	33	31	31			
C_8mo_						
GM (%CV)	0.43 (34.8)	0.37 (35.9)	0.26 (41.2)	1.14 (0.92, 1.42)	1.65 (1.17, 2.32)	1.44 (1.01, 2.06)
P_10_ - P_90_*	0.25 - 0.66	0.24 - 0.63	0.18 - 0.49			
% < 0.2 ng/mL	3.3	7.4	12.0			
Total	30	27	25			
C_12mo_						
GM (%CV)	0.48 (29.4)	0.41 (29.6)	0.32 (43.0)	1.17 (0.98, 1.39)	1.49 (1.15, 1.94)	1.28 (0.97, 1.67)
P_10_ - P_90_*	0.30 - 0.72	0.25 - 0.58	0.20 - 0.57			
% < 0.2 ng/mL	0.0	0.0	13.6			
Total	29	22	22			

⁎ P_10_ and P_90_ are the 10th and 90th percentiles respectively.

**Figure 3:**
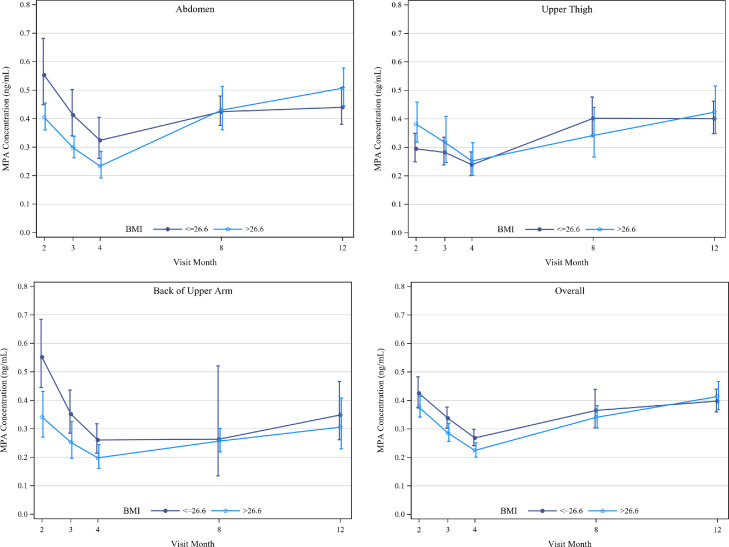
Geometric Mean Medroxyprogesterone Acetate (MPA) Concentrations by Median Body Mass Index (BMI) with 90% Confidence Intervals (slightly shifted for visibility) summarized among women in the PK Population. Median BMI was calculated using data from all three injection sites. Summaries provided for women receiving injections A) in the Abdomen, B) in the Upper Thigh, C) in the Back of Upper Arm, and D) Overall.

The month 12 trough AR [95% CI] was similar for the abdomen (1.76 [1.43, 2.17]) and thigh (1.70 [1.36, 2.13]), but noticeably lower for the arm (1.39 [1.06, 1.84]). Modeling the month 2, 3, and 4 data yielded apparent half-life estimates of 77 days, 143 days, and 63 days, respectively, for the abdomen, thigh, and arm. Neither study center nor age significantly impacted PK of MPA. Overall, there were no statistically significant differences in GM concentrations by BMI over time, though trends by BMI differed for the thigh compared to the abdomen and arm (Figure 3).

### Safety

Seventy-three participants (9.7%) experienced reportable AEs. Two serious adverse events were reported; one participant experienced a stroke considered possibly related and one experienced a leg fracture considered not related to the study product. The most frequently occurring reportable adverse events were bleeding irregularities (her cycle length, frequency and/or volume varied from what was normal for her prior to using Sayana Press), reported by 39 (5.2%) participants.

The frequency of amenorrhea increased during follow-up: the proportion of women not having menstrual-like bleeding since their last injection was 24.9%, 42.8% and 52.3% at the months 4, 8 and 12 visits, respectively. There was a trend toward decreased participant reporting of heavy bleeding from baseline throughout follow-up (17.2% baseline, 8.1% month 4, 6.7% month 8, 9.4% month 12).

Eighty (10.7%) women experienced 126 ISRs (among a total of 2028 injections administered in the study), among which only 17 (13.5%) were of Grade 1 (or mild) severity and documented as adverse events. The most frequently reported ISR types were hypopigmentation at the site of injection (66 occurrences experienced by 44 (5.9%) women) and atrophy (54 occurrences experienced by 30 (4.0%) women). Twenty out of 44 women with hypopigmentation had more than one occurrence and 18 out of 30 women with atrophy had more than one occurrence. Out of 59 women who had hypopigmentation or atrophy 15 had both. Both hypopigmentation and atrophy occurred more frequently among women receiving injections in the thigh (8.5% and 3.9%, respectively) as compared to the abdomen (7.6% and 0.8%, respectively). Twenty-five (37.8%) hypopigmentation events were reported by participants and 41 (62.1%) were identified by study staff; the majority (98.5%) occurred one month after injection or later. All except one (98%) hypopigmentation events were noted in participants who self-identified as bi- or multi-racial. Redness (15 occurrences experienced by 13 (1.7%) women) and pain (11 occurrences experienced by 10 (1.3%) women) were reported infrequently.

The median change in weight from baseline to the month 12 visit was 3 kgs (25th percentile 0 kg, 75th percentile 6 kg) and 69.4% of participants who reported weight at month 12 experienced weight gain. The median change in systolic and diastolic blood pressure were 0 and 1 mmHg (range −34 to 42 and −27 to 37) respectively

### Acceptability

Method acceptability was high among 702 women who responded to questionnaires at their final visit; 90.2% (*n* = 632/701, one woman did not answer this question) of participants reported being satisfied or very satisfied with Sayana Press. Ninety three percent (*n* = 653) reported that they would use Sayana Press as a 4-month method in the future if found to be effective, 97.3% (*n* = 683) stated they would recommend the method to a friend or family member and most women (74.5%; *n* = 523) also stated that they would be willing to self-inject if provided with instructions.

## Discussion

We restricted the primary effectiveness analysis to 682 women receiving injections in the abdomen and thigh because those are the sites listed in the Sayana Press package leaflet.^18^ Our results confirm that Sayana Press, and by extension Depo-subQ Provera 104, is a highly effective contraceptive method when administered every 4 months at those sites. No pregnancies were observed, resulting in an estimated Pearl Index of 0.00 pregnancies per 100 WY of use (95% CI 0.00, 0.59).

Pfizer's development program for Depo-subQ 104 presumed that MPA levels exceeding 0.2 ng/mL were necessary to reliably suppress ovulation. In our study, however, 8.8%, 22.6%, and 16.1% of women receiving injections in the abdomen, thigh, and arm, respectively, were below 0.2 ng/mL at month 3, and approximately 10% in each injection site were below 0.14 ng/mL at month 4. The absence of any pregnancies strongly supports the theory that the contraceptive threshold of MPA is lower than 0.2 ng/mL, and that the 0.1 ng/mL level noted in other studies^10^^,^^19^ is a more reasonable guidance.

Somewhat lower MPA levels at months 4, 8 and 12 among women receiving injections in the arm are consistent with previous findings^20^ and in theory could translate into a higher risk of ovulation for that injection site. Whether a hypothetical higher risk of ovulation due to lower average trough levels translates into a meaningfully higher risk of pregnancy is not clear. There were no pregnancies among 40 women who received Sayana Press injection in the arm in our study, although these data are insufficient in and of themselves for a definitive conclusion due to the small numbers. Importantly, we observed significant accumulation of MPA over multiple injection cycles (76%, 70%, and 39% higher estimated geometric mean levels at month 12 than month 4 for the abdomen, thigh, and arm, respectively; Table 3). Hence, any potential risk of pregnancy that may exist in the first injection cycle likely diminishes over time, regardless of injection site. Exploratory analyses also found that MPA levels may be somewhat lower among women with higher BMI during the first injection cycle, but the effect was not evident by month 12.

Our attempt to estimate the apparent half-life in the first injection cycle relied on sparse timepoints and was inconsistent (too low) with the observed level of accumulation for the abdomen. A phase 1 PK study comparing subcutaneous injection of Sayana Press in the upper arm versus the thigh and abdomen is in planning by the manufacturer which may provide greater insight.^21^

Adverse event data collection was limited, but reported events suggest similar side effects frequently associated with injectable contraceptives, the most common of which was bleeding disturbances. Similar to previous reports, the proportion of women with amenorrhea increased with increased duration of use of SP.^9^ Most ISRs reported in the study are consistent with the Sayana Press package insert, except for hypopigmentation. In our study, hypopigmentation was the most reported ISR and was experienced predominantly by participants who identified as bi- or multi-racial. Our data also indicate that some women may be more prone to hypopigmentation, atrophy, or both. Approximately half of women with hypopigmentation or atrophy in our study had more than one occurrence, and 25% had both. It is unclear why none of the previous studies reported hypopigmentation as a possible side effect of subcutaneous DMPA. One possible explanation is that the reaction is somewhat delayed after injection and may not be observed and/or reported by providers and women. Another possible explanation is racial composition of our study population; our study had a high proportion (62%) of bi- or multi-racial participants and hypopigmentation may be more noticeable and/or may occur more frequently, among individuals with darker skin tone. In addition, we evaluated injection sites for possible reactions systematically throughout the study so the higher rate of ISRs compared to the data from prior studies may be due to the ascertainment bias. The more frequent occurrence of ISRs in the thigh vs. the abdomen is consistent with previous reports.^15^

Clinical staff reported difficulty with the Uniject device in a limited number of cases early during the trial; thereafter, Uniject device issues were systematically captured on study case report forms. The frequency of device blockage declined over time; anecdotally, clinical staff reported that increased duration of shaking prevented blockage.

Important strengths of the study include minimal loss to follow-up and high injection schedule adherence (95.7% of injections were administered on target or in the grace period). Moreover, participants reported vaginal sex in over 99% of treatment cycles and condom use in only 11% of treatment cycles, confirming that the population was at risk of pregnancy, increasing validity of our data. The study also enrolled an ethnically diverse sample of reproductive aged women in Latin America that supports generalization of the results more broadly. By design, the trial was unable to evaluate Sayana Press effectiveness when injected every 4 months in the upper arm. While hypopigmentation was the mostly frequently reported ISR, our data collection methods were not designed to characterize these events in more detail and accurately estimate their time of onset or duration. In addition, clinical staff were unblinded to injection site, which could have influenced assessment of secondary outcomes. Regarding PK analyses, exclusion of the 20 participants due to unevaluable baseline MPA specimens, baseline MPA concentrations >0.05 ng/mL or use of a second device due to Uniject malfunction resulted in a lower number of participants than planned for PK analyses and may have lowered our statistical power to detect relative differences in PK parameters between injection sites. Lastly, it is possible that participants reporting of method acceptability was influenced by the face-to-face interview data collection method.

Our data support 4-monthly administration of Sayana Press when injected in the abdomen and thigh, independent of BMI, and likely independent of injection site, although data in the upper arm are limited. Administration of Sayana Press every 4 months would decrease user and health system costs, thus making the product available to more women in need of effective contraception. Amending the current product label to extend the duration of action to 4 months with a 1-week reinjection window would ensure use of a lower effective dose and reduce women's long-term MPA exposure. At a minimum, our data clearly support amending the Sayana Press product use instructions to extend the duration of the reinjection window from the current one week^22^ to four weeks and reinforce the World Health Organization Selected Practice Recommendations (WHO SPR) that already allow for a 4-week reinjection window for all DMPA injectables.^23^ A formal change in the Sayana Press labeling would be an important step towards increasing access to the product, especially in countries where Ministries of Health do not widely rely on or utilize WHO SPR guidelines.

## Author contributions

DT, VH and LD designed the trial. RF and DT analyzed the data; RF and Lalitha Venkatasubramanian verified the data. JD provided overall leadership of trial conduct; VB, LB and AS led trial conduct at Profamilia, UNICAMP and ICMER, respectively. All authors contributed equally to results interpretation. JD drafted the manuscript, and all authors approved the final version.

## Funding

This work is made possible by the generous support of the American people through the U.S. Agency for International Development (USAID), provided to FHI 360 through Cooperative Agreement AID-OAA-A-15-00045, and a grant from the Gates Foundation. The contents are the responsibility of FHI 360 and do not necessarily reflect the views of USAID, the United States Government, or the Gates Foundation, nor does any mention of trade names, commercial products, or organizations imply endorsement by FHI 360, USAID, the United States Government, or the Gates Foundation.

## Declaration of interests

LD and NV report grants from the Bill & Melinda Gates Foundation and USAID (during the conduct of the study). JD reports grants through a USAID cooperative agreement paid directly to the institution (during the conduct of the study). AS reports membership on the Chilean Society of Reproductive Medicine (SOCMER) Board of Directors (in the past 36 months). VH reports funding from USAID for technical assistance and medical writing (during conduct of the study). All other authors declare no competing interests.

## Supplementary materials

Image, application 1
