# Supplementary material for: Contraceptive effectiveness, pharmacokinetics, and safety of Sayana® Press when injected every four months: a multicenter phase 3 trial
Source: eClinicalMedicine. 2022 Jan 29;44:101273. doi: 10.1016/j.eclinm.2022.101273 (PMC8804165; doi:10.1016/j.eclinm.2022.101273)

CLINICAL STUDY PROTOCOL

**Title:** A Study to Evaluate the Effectiveness, Pharmacokinetics, Safety, and Acceptability of Sayana® Press when Injected Every Four Months

**FHI 360 Study #:** 926400

**Version:** 8.0-B (for Brazil site participating in optional specimen collection for the Vaginal Immunity Study, optional DXA scan for the Body Composition and Bone Mineral Density Study, and Extended Follow-up Subset)

**Funded by:** USAID

The Bill & Melinda Gates Foundation

**Investigational Drug:** Sayana® Press (medroxyprogesterone acetate injectable suspension, 104 mg/0.65 mL)

**Study Sponsor:** FHI 360

359 Blackwell Street, Suite 200

Durham, NC 27701

**Study Director:** Jennifer Deese, PhD, MPH, CCRP

Scientist

FHI 360

359 Blackwell Street, Suite 200

Durham, NC 27701

Tel: 1-919-544-7040 x 11292

[jdeese@fhi360.org](mailto:jdeese@fhi360.org)

**Site investigators and Sites:** Lic. Vivian Brache

Asociación Dominicana Pro Bienestar de la Familia, Inc. (PROFAMILIA)

Nicolas de Ovando esq. Calle 16

Ens. Luperon

Santo Domingo, Dominican Republic

Tel: 1-809-681-8357

[vbrache@gmail.com](mailto:vbrache@gmail.com)

Luis Bahamondes, MD

Family Planning Clinic

Department of Obstetrics and Gynecology

Faculty of Medical Sciences

Universidade Estadual de Campinas (UNICAMP)

Caixa Postal 6181

13084-971 Campinas, SP, Brazil

Tel: 55-19-3289-2856

[drluisbahamondes@hotmail.com](mailto:drluisbahamondes@hotmail.com)

Abril Salinas Quero, MD

Instituto Chileno De Medicina Reproductiva (ICMER)

Jose Victorino Lastarria 29, ap 101

Santiago, Chile

Tel: 56- 968423737

[abrilsalinasq@gmail.com](mailto:abrilsalinasq@gmail.com)

**Medical Monitor:** Vera Halpern, MD

Director, Medical Research

FHI 360

Tel: 1-919-544-7040 x 11390

[vhalpern@fhi360.org](mailto:vhalpern@fhi360.org)

**Central Laboratory:** PPD Development

2244 Dabney Road

Richmond, VA 23230

Tel: 1-804-359-1900

This protocol is the confidential information of FHI 360 and is intended solely for the guidance of the clinical investigation. This protocol may not be disclosed to parties not associated with the clinical investigation or used for any purpose without the prior written consent of FHI 360.

# INVESTIGATOR SIGNATURE FORM

**A Study to Evaluate the Effectiveness, Pharmacokinetics, Safety, and Acceptability of Sayana® Press when Injected Every Four Months**

**Version: 8.0-B**

**Date: 23 January 2020**

**Funded by: USAID and The Bill & Melinda Gates Foundation**

I, the site investigator, agree to conduct this study in full accordance with the provisions of this protocol and in accordance with the International Conference on Harmonization (Section E6 (R1) Good Clinical Practice), US Department of Health and Human Services 45 Code of Federal Regulations Part 46, local regulatory requirements, and the Statement of Investigator, which I have also signed. I agree to maintain all study documentation until FHI 360 advises that it is no longer necessary.

I read and understood the information in this protocol and the Prescribing Information for the clinical product used in this study, including the potential risks and side effects and will ensure that all associates, colleagues, and employees assisting in the conduct of the study are informed about the obligations incurred by their contribution to the study.

____________________________

Printed name of Site Investigator

____________________________ ______________________________

Signature of Site Investigator Date

# CLINICAL STUDY SYNOPSIS

**Title of Protocol:** A Study to Evaluate the Effectiveness, Pharmacokinetics, Safety, and Acceptability of Sayana® Press when Injected Every Four Months

**Study Sponsor:** FHI 360

**Funded by:** USAID

The Bill & Melinda Gates Foundation

**Study Number**: 926400

**Investigational Drug:** Sayana® Press (medroxyprogesterone acetate injectable suspension, 104 mg/0.65 mL)

**Active Ingredient:** Medroxyprogesterone acetate (MPA)

**Phase of Study:** 3

**Number of Sites:** 3

**Countries Planned:** Brazil, Chile, and Dominican Republic

**Study Duration:** Recruitment is expected to take 9 months and each participant will be followed for 12 months of product use. A subset of participants will be followed until resumption of ovulation or up to a maximum of 12 months from the last study injection. The total study duration is anticipated to be 41 months, including initiation activities, analysis, and reporting.

**Study Population:** A total of 750 healthy, sexually active women between 18 and 35 years of age who are willing to use Sayana® Press injected every 4 months as their only means of contraception for 12 months.

**Primary Objective:** To evaluate the effectiveness of Sayana® Press injected subcutaneously every 4 months in the abdomen or upper thigh for 12 months (3 treatment cycles) of use.

**Primary Endpoint:** Pregnancy

**Secondary Objectives:** 1) To assess trough concentrations, accumulation and apparent terminal half-life of MPA, and the impact of subcutaneous injection site (abdomen, upper thigh, or upper arm) on these parameters, when Sayana® Press is injected every 4 months for 12 months of use;

2) To evaluate the safety of Sayana® Press when injected every 4 months for 12 months of use;

3) To evaluate the acceptability of Sayana® Press when injected every 4 months for 12 months of use.

**Secondary Endpoints:** Serum MPA concentrations on day 0 (baseline), months 2, 3, 4, 8, and 12 after treatment initiation (subset of 120 participants, only); serious adverse events and adverse events leading to product withdrawal; injection site reactions, bleeding patterns, blood pressure, and body weight; and responses to acceptability questions.

**Exploratory Objective:** To evaluate return to ovulation among a subset of study participants who received month 4 and month 8 injections and plan to use non-hormonal methods of contraception, or no contraception, for up to a maximum of 12 months from the last study injection.

**Exploratory Endpoint:** Return to ovulation, where ovulation is defined as a single elevated serum progesterone (P ≥4.7 ng/mL) or a confirmed pregnancy test.

**General Design and Methodology:**

This study will evaluate the effectiveness, pharmacokinetics (PK), safety, and acceptability of Sayana® Press when injected every 4 months (17-18 weeks) for 3 treatment cycles (12 months) of use. A total of 750 healthy, sexually active women aged 18 to 35 years with regular menstrual cycles and no DMPA use in the previous 12 months will be enrolled and followed for pregnancy. Among all enrolled, 710 will be randomized to receive injections in the abdomen or upper thigh in accordance with the PATH Sayana® injection instructions (Appendix 1), which are consistent with prescribing information, for the primary effectiveness analysis. An additional 40 women will be randomized to receive injections subcutaneously in the back of the upper arm to assess whether differences in PK may exist which could impact the grace period for reinjections for that injection site.

Treatment will begin in the first 5 days of menses. A single MPA serum sample will be collected from all participants at baseline. All participants will receive re-injections at month 4 (17 weeks after treatment initiation) and month 8 (35 weeks after treatment initiation), and complete their scheduled follow-up at approximately month 12 (53 weeks after treatment initiation). There will be a plus 7-day grace period for re-injections, but women who are up to 28 days late may continue treatment if they have a negative urine pregnancy test. Urine pregnancy testing will be performed for all women at enrollment, at month 4, at month 12/study exit and at any other time during the study if clinically indicated. An additional urine pregnancy test will be completed at month 8 for Brazilian women enrolled in the Body Composition and Bone Mineral Density (BC-BMD) sub-study. Participants will be evaluated for injection site reactions and asked to provide information on adverse events, prohibited concomitant medication and other contraceptive use, vaginal bleeding, and method acceptability at months 4, 8 and 12; information on adverse events and prohibited concomitant medications will be recorded if self-reported by participants at the month 2 and 3 PK visits, and at unscheduled visits.

**Method of Randomization:**

One cohort of N=630 will be randomized 1:1 to receive their subcutaneous injections of Sayana® Press in the abdomen or upper thigh, and a second PK cohort of N=120 women who agree to serum MPA testing at 6 time points (baseline and months 2, 3, 4, 8, and 12) will be randomized 1:1:1 to receive their injections in the abdomen, upper thigh, or back of the upper arm. Using this randomization scheme, 355 women will receive their injections in the abdomen, 355 in the upper thigh, and 40 in the back of the upper arm. Randomization in each cohort will use randomly-permuted blocks, stratified by research center. Neither participants nor site staff will be blinded.

**Study Drug Dose and Mode of Administration:**

Sayana® Press (MPA injectable suspension, 104 mg/0.65 mL), injected subcutaneously in the abdomen, upper thigh, or back of the upper arm using the pre-filled Uniject^TM^ delivery device.

**Criteria for Inclusion:**

Women may be included in the study if, at enrollment, they meet all of the following criteria:

- - not pregnant and no desire to become pregnant in the next 18 months
  - regular menstrual cycles (25 to 35 days in length when not using hormonal contraception, pregnant, or lactating)
  - at risk of pregnancy (no diagnosis of infertility, no history of tubal ligation or hysterectomy, and an average of 1 or more unprotected acts of vaginal intercourse per month)
  - in good general health as determined by a medical history
  - 18 to 35 years of age, inclusive
  - willing to provide informed consent, follow all study requirements, and rely on Sayana® Press injected every 4 months as the only means of contraception for 12 months
  - has only one sexual partner and expects to have that same sexual partner for the next 12 months

**Criteria for Exclusion:**

Women will be excluded from this study if they meet any of the following criteria at enrollment:

- has a primary partner who has received a vasectomy or is otherwise sterile
- medical contraindications to depot medroxyprogesterone acetate (DMPA) per World Health Organization medical eligibility criteria for contraceptive use
- known HIV-infection (for her or her partner)
- diagnosis or treatment for a sexually transmitted infection in the past month (for her or a partner), excluding recurrent herpes or condyloma
- received an injection of a progestin-only containing contraceptive (DMPA or norethisterone enanthate) in the past 12 months
- received an injection of a combined injectable contraceptive in the past 6 months
- known or suspected allergic reaction to DMPA
- used a levonorgestrel-releasing intrauterine system, NuvaRing, contraceptive patch, oral contraceptives or a contraceptive implant in the 7 days prior to enrollment (PK cohort only)
- previous (within 1 month prior to enrollment), current or planned (in the next 12 months) use of an investigational drug, prohibited drug per protocol or other drug which in the opinion of the site investigator could complicate study findings
- has been pregnant in the past month
- is lactating
- plans to move to another location in the next 12 months
- has a social or medical condition which in the opinion of the site investigator would make study participation unsafe, or interfere with adherence to protocol requirements.

**Study size and Statistical Analysis:**

The 710 women receiving injections in the abdomen or upper thigh will constitute the primary efficacy population. This study size is sufficient to ensure that the difference between the estimated Pearl Index (pregnancies per 100 woman-years of use) and the corresponding upper 95% confidence bound does not exceed 1.0, so long as the observed index is less than 0.75 and at least 80% of participants complete 12 months of follow-up. The PK cohort of 120 women (40 receiving injections in each the abdomen, upper thigh, and back of the upper arm) is sufficient to provide 85% power to detect 30% relative differences in PK parameters between injection sites, assuming the coefficient of variation is no more than 40% and using two-sided 0.05 significance tests.

**Interim analysis:**

An independent Data and Safety Monitoring Board (DSMB) will review interim data when 250 participants have completed 4 months of follow-up. This single, pre-planned interim analysis is intended to inform a decision whether to stop or modify the study (e.g., drop an injection site) if the estimated probability of pregnancy is greater than 2% in the first injection interval. Additional interim analyses may be requested by the DSMB based on periodic reviews of study indicators (pregnancy, adverse event, recruitment and continuation rates) if deemed necessary to protect participant safety or to make recommendations to modify the study.

TABLE OF CONTENTS

[INVESTIGATOR SIGNATURE FORM 3](#_Toc28678394)

[CLINICAL STUDY SYNOPSIS 4](#_Toc28678395)

[LIST OF ABBREVIATIONS 13](#_Toc28678396)

[1 BACKGROUND INFORMATION 14](#_Toc28678397)

[1.1 Introduction 14](#_Toc28678398)

[1.2 Name and Description of Product 15](#_Toc28678399)

[1.3 Findings from Nonclinical and Clinical Studies 15](#_Toc28678400)

[1.3.1 Nonclinical Studies 15](#_Toc28678401)

[1.3.2 Clinical Studies 16](#_Toc28678402)

[1.4 Known and Potential Risks and Benefits to Human Participants 19](#_Toc28678403)

[1.4.1 Known and Potential Health Risks of the Study Drug 19](#_Toc28678404)

[1.4.2 Benefits to Participants 21](#_Toc28678405)

[1.5 Selection of Drugs and Dosages 21](#_Toc28678406)

[1.6 Compliance Statement 21](#_Toc28678407)

[1.7 Population to Be Studied 22](#_Toc28678408)

[2 PURPOSE OF THE STUDY AND STUDY OBJECTIVES 22](#_Toc28678409)

[2.1 Purpose of the Study 22](#_Toc28678410)

[2.2 Study Objectives 22](#_Toc28678411)

[2.2.1 Primary Objective 22](#_Toc28678412)

[2.2.2 Secondary Objectives 22](#_Toc28678413)

[2.2.3 Exploratory Objective 22](#_Toc28678414)

[2.3 Study Endpoints 23](#_Toc28678415)

[2.3.1 Primary Endpoint 23](#_Toc28678416)

[2.3.2 Secondary Endpoint 23](#_Toc28678417)

[2.3.3 Exploratory Endpoint 23](#_Toc28678418)

[3 STUDY DESIGN 23](#_Toc28678419)

[3.1 General Design and Study Schema 23](#_Toc28678420)

[3.2 Justification for the Study Design 25](#_Toc28678421)

[3.3 Efficacy Measure 25](#_Toc28678422)

[3.4 Pharmacokinetic Measures 26](#_Toc28678423)

[3.5 Safety Measures 26](#_Toc28678424)

[3.6 Acceptability Measures 26](#_Toc28678425)

[3.7 Randomization, Allocation Concealment and Blinding 26](#_Toc28678426)

[3.7.1 Randomization 26](#_Toc28678427)

[3.7.2 Allocation Concealment 26](#_Toc28678428)

[3.7.3 Blinding 26](#_Toc28678429)

[3.8 Data and Safety Monitoring Board 27](#_Toc28678430)

[3.9 Study Drug and Dosage 27](#_Toc28678431)

[3.10 Drug Supply and Accountability 27](#_Toc28678432)

[3.10.1 Drug Accountability 27](#_Toc28678433)

[3.11 Duration of Study Participation 28](#_Toc28678434)

[3.12 Discontinuation Criteria 28](#_Toc28678435)

[3.12.1 Study Termination 28](#_Toc28678436)

[3.12.2 Participant Discontinuation 28](#_Toc28678437)

[3.12.3 Drug Supply, Storage and Security 28](#_Toc28678438)

[3.13 Source Data Recorded on Case Report Forms 28](#_Toc28678439)

[3.14 Study Procedures 29](#_Toc28678440)

[3.14.1 Screening Visit 30](#_Toc28678441)

[3.14.2 Enrollment Visit (Day 0) 31](#_Toc28678442)

[3.14.3 Regularly Scheduled Follow-up Visits 32](#_Toc28678443)

[3.14.4 Additional Blood Draw Visits (Months 2 and 3) 33](#_Toc28678444)

[3.14.5 Additional Vaginal, Endocervical and Blood Samples 34](#_Toc28678445)

[3.14.6 Additional BC and BMD Measurements 34](#_Toc28678446)

[3.14.7 Extended Follow-Up Subset Procedures 34](#_Toc28678447)

[3.14.8 Missed and Unscheduled Visits 34](#_Toc28678448)

[3.14.9 Post-Discontinuation Visits 35](#_Toc28678449)

[3.14.10 Loss to Follow Up 35](#_Toc28678450)

[4 SELECTION AND WITHDRAWAL OF PARTICIPANTS 35](#_Toc28678451)

[4.1 Inclusion Criteria 35](#_Toc28678452)

[4.2 Exclusion Criteria 35](#_Toc28678453)

[4.3 Justification for Key Inclusion and Exclusion Criteria 36](#_Toc28678454)

[4.4 Withdrawal Criteria and Procedures 36](#_Toc28678455)

[5 TREATMENT OF PARTICIPANTS 37](#_Toc28678456)

[5.1 Study Drugs Administered During the Study 37](#_Toc28678457)

[5.2 Restrictions 37](#_Toc28678458)

[5.3 Prior and Concomitant Therapy or Medication 37](#_Toc28678459)

[5.4 Procedures for Monitoring Participant Treatment Compliance 37](#_Toc28678460)

[6 ASSESSMENT OF EFFICACY 37](#_Toc28678461)

[7 ASSESSMENT OF PHARMACOKINETICS 38](#_Toc28678462)

[7.1 Pharmacokinetic Measures 38](#_Toc28678463)

[7.2 Blood Sampling, Handling, and Laboratory Analysis 38](#_Toc28678464)

[8 ASSESSMENT OF SAFETY 38](#_Toc28678465)

[8.1 Adverse Events 39](#_Toc28678466)

[8.2 Serious Adverse Events 39](#_Toc28678467)

[8.2.1 Definition of a Serious Adverse Event 39](#_Toc28678468)

[8.2.2 Reporting SAEs 40](#_Toc28678469)

[8.3 Injection site reactions 40](#_Toc28678470)

[8.4 Return to ovulation (extended follow-up subset only) 40](#_Toc28678471)

[8.4.1 Blood Sampling, Handling, and Laboratory Analysis 41](#_Toc28678472)

[8.5 Pregnancy outcomes 41](#_Toc28678473)

[8.6 Bleeding Assessment 42](#_Toc28678474)

[8.7 Measurement of Body weight and BP 42](#_Toc28678475)

[8.8 Concomitant Medication 42](#_Toc28678476)

[8.9 Social Harm Events 42](#_Toc28678477)

[8.10 Methods and Timing of Assessing, Recording, and Analyzing Safety Data 42](#_Toc28678478)

[9 ASSESSMENT OF ACCEPTABILITY 42](#_Toc28678479)

[10 STATISTICS 42](#_Toc28678480)

[10.1 Sample Size and Power Considerations 42](#_Toc28678481)

[10.2 Analysis Populations and Analysis Sets 43](#_Toc28678482)

[10.2.1 Treated Population and Analysis Set 43](#_Toc28678483)

[10.2.2 Pharmacokinetics Population and Analysis Set 43](#_Toc28678484)

[10.2.3 Extended Follow-up Population 43](#_Toc28678485)

[10.3 Data Handling Conventions 43](#_Toc28678486)

[10.4 Multiple Comparisons and Multiplicity 44](#_Toc28678487)

[10.5 Assessment of Study Populations 44](#_Toc28678488)

[10.5.1 Participant Disposition 44](#_Toc28678489)

[10.5.2 Demographic and other Baseline Characteristics 44](#_Toc28678490)

[10.6 Efficacy Analysis 44](#_Toc28678491)

[10.6.1 Primary Endpoint 44](#_Toc28678492)

[10.6.2 Planned Methods of Analysis 45](#_Toc28678493)

[10.7 Pharmacokinetic Analysis 45](#_Toc28678494)

[10.7.1 Pharmacokinetic Endpoints 45](#_Toc28678495)

[10.7.2 Planned Method of Analysis 45](#_Toc28678496)

[10.8 Safety Endpoints and Analysis 46](#_Toc28678497)

[10.8.1 Safety Endpoints 46](#_Toc28678498)

[10.8.2 Safety Analysis 46](#_Toc28678499)

[10.9 Acceptability Variables and Analysis 46](#_Toc28678500)

[10.10 Exploratory Variables and Analysis 46](#_Toc28678501)

[10.11 BC and BMD Variables and Analysis 46](#_Toc28678502)

[10.12 Planned Interim Analysis 47](#_Toc28678503)

[10.13 Reporting Deviations from the Statistical Plan 47](#_Toc28678504)

[11 DIRECT ACCESS TO SOURCE DATA/DOCUMENTS 47](#_Toc28678505)

[12 QUALITY CONTROL AND QUALITY ASSURANCE 47](#_Toc28678506)

[12.1 Protocol Amendments 47](#_Toc28678507)

[12.2 Protocol Violations 48](#_Toc28678508)

[12.3 Information to Study Personnel 48](#_Toc28678509)

[12.4 Study Monitoring 48](#_Toc28678510)

[12.5 Audit and Inspection 49](#_Toc28678511)

[13 ETHICS 49](#_Toc28678512)

[13.1 Informed Consent 49](#_Toc28678513)

[13.2 Compensation 49](#_Toc28678514)

[13.3 Health Authorities and IRBs 50](#_Toc28678515)

[13.4 Study Participant Confidentiality 50](#_Toc28678516)

[13.5 Registration of the Clinical Study 50](#_Toc28678517)

[14 DATA HANDLING, DATA QUALITY CONTROL, AND RECORD KEEPING 50](#_Toc28678518)

[14.1 Data Collection 50](#_Toc28678519)

[14.2 Data Quality Control 51](#_Toc28678520)

[14.3 Archiving of Case Report Forms and Source Documents 51](#_Toc28678521)

[14.3.1 Investigator Responsibilities 51](#_Toc28678522)

[14.3.2 FHI 360 Responsibilities 52](#_Toc28678523)

[15 FINANCING AND INSURANCE 52](#_Toc28678524)

[16 REPORTING AND PUBLICATION OF RESULTS 52](#_Toc28678525)

[17 REFERENCES 53](#_Toc28678526)

[18 APPENDICES 55](#_Toc28678527)

[18.1 Appendix 1: Sayana^®^ Press Injection Job Aid 55](#_Toc28678528)

**LIST OF ABBREVIATIONS**

AE Adverse Event

BC Body Composition

BC-BMD Body Composition and Bone Mineral Density Study

BP Blood pressure

BMD Bone Mineral Density

C_xmo_ Serum MPA Concentration at Month ‘x’ following Injection

CDMS Clinical Data Management System

CRF Case Report Form

DSMB Data and Safety Monitoring Board

DMPA Depot Medroxyprogesterone Acetate

DXA Dual Energy X-ray absorptiometry (DXA)

EDF Estimated Date of Fertilization

FN Femoral neck

FSH Follicle Stimulating Hormone

GCP Good Clinical Practice

hCG Human Chorionic Gonadotropin

HIV Human Immunodeficiency Virus

ICH International Council for Harmonization

IM Intramuscular

IRB Institutional Review Board

ISR Injection Site Reaction

LMP Last Menstrual Period

LS Lumbar spine

LH Luteinizing Hormone

MPA Medroxyprogesterone Acetate

NDA New Drug Application

PD Pharmacodynamics

PK Pharmacokinetics

PHSC Protection of Human Subjects Committee

SAE Serious Adverse Event

SAP Statistical Analysis Plan

SC Subcutaneous

SOP Standard Operating Procedure

STI Sexually Transmitted Infection

T_1/2_ Apparent Terminal Half-Life

USAID United States Agency for International Development

USFDA United States Food and Drug Administration

WHO World Health Organization

WY Woman-Years

# BACKGROUND INFORMATION

## Introduction

Globally, over 40 million women use injectable contraceptives for pregnancy prevention [[1](#_ENREF_1)]. Depo-Provera® CI (150 mg/mL medroxyprogesterone acetate [MPA] injectable suspension) (Pfizer, New York, NY) and generic versions of the same are the most commonly used injectable method. Also known as depot medroxyprogesterone acetate (DMPA) for intramuscular (IM) administration (DMPA-IM), it has a decades-long history as a highly efficacious contraceptive, with a pregnancy rate of approximately 0.3 per 100 woman-years (WY) when injected as prescribed at a dose of 150 mg every 3 months [[2](#_ENREF_2)]. The World Health Organization’s (WHO) evidence-based family planning guidance further specifies that repeat injections can be given up to 4 weeks late without requiring additional contraceptive protection [[3](#_ENREF_3)]. Although widely used, DMPA-IM carries a burden of side effects including weight gain, bleeding disturbances, metabolic effects, reduced bone mineral density (BMD) [[4-6](#_ENREF_4)] and delayed return to fertility [[7](#_ENREF_7)]. The use of DMPA-IM has also been associated with an increased risk of Human Immunodeficiency Virus (HIV) acquisition [[8](#_ENREF_8), [9](#_ENREF_9)], but a causal relationship has not been established.

More recently, Pfizer developed an alternative MPA formulation for subcutaneous (SC) injection in the abdomen or upper thigh (Depo-SubQ Provera 104®). Due in part to a slower rate of absorption following SC administration [[10](#_ENREF_10)], Depo-SubQ Provera 104® achieves the same degree of efficacy as DMPA-IM but at a 31% lower dose (104 mg/0.65 mL every 3 months) [[11](#_ENREF_11), [12](#_ENREF_12)]. Because of the ease of SC administration, Depo-SubQ Provera 104® and in particular Sayana® Press (0.65 mL of Depo-SubQ Provera 104® pre-filled in the UnijectTM delivery system) have the potential to increase access in settings where resource constraints can be a barrier to provision and consistent use of injectable contraception. Studies have demonstrated that it can be safely provided outside the clinic setting (e.g., using community health workers) and that self-injection is acceptable and feasible [[13](#_ENREF_13), [14](#_ENREF_14)]. Study findings have motivated donors such as the U.S. Agency for International Development (USAID) to try to expand access to Sayana® Press in developing countries, but price remains a barrier.

Despite its 31% lower dose, side effects of Depo-SubQ Provera 104® remain similar to DMPA-IM [[11](#_ENREF_11)]. This could be related to the slower release rate for the SC product, resulting in a similar steady-state trough MPA concentration as DMPA-IM; in a randomized study, average serum concentrations 1 and 2 years after initiating Depo-SubQ Provera 104® were 100% and 85% of the corresponding DMPA-IM values [[12](#_ENREF_12)]. The potential dose-dependent nature of some side effects has motivated interest in lowering the MPA exposure still further [[15](#_ENREF_15)]. Indeed, the lack of any pregnancies in phase 3 trials of Depo-SubQ Provera 104® [[16](#_ENREF_16)] is consistent with the hypothesis that the injected dose is higher than necessary.

We hypothesize that a high degree of contraceptive efficacy will be maintained when extending the injection interval of Sayana® Press from 3 to 4 months. Extending the interval would reduce long-term MPA exposure by 25% and lower steady state trough MPA concentrations, thus shortening the delay in return to fertility and possibly reducing the severity of other dose-dependent side effects. This would also decrease programmatic costs, thereby facilitating access to Sayana® Press in resource-constrained settings. We base our hypothesis on a review of available pharmacokinetics (PK) and pharmacodynamics (PD) data for Depo-SubQ Provera 104®. This review suggests that the risk of ovulation following initiation of Sayana® Press might be minimal, and that the accumulation of MPA (and hence the duration of action following multiple doses) is likely greater than was reported in the main PK study that informed the regulatory approval of Depo-SubQ Provera 104®[[10](#_ENREF_10)]. In order to test our hypothesis, we will conduct a clinical study to estimate the effectiveness of Sayana® Press when injected every 4 months for 12 months (3 injection cycles) of use. We will also estimate trough MPA concentrations and apparent terminal half-life to determine whether the anatomical site of injection meaningfully affects the PK of MPA, and to inform an appropriate grace period for re-injections outside the clinical trial setting where the dosing interval is tightly controlled.

One of the important dose-dependent side effects of the existing 3-month DMPA is delayed return to ovulation. The 4-month re-injection interval may improve the side effect profile, specifically shortening time to return to ovulation, by lowering the overall drug exposure. We will collect limited clinical data from a subset of women participating in this study on return to ovulation. This analysis can help to inform how much sooner women may expect to return to ovulation (and therefore, to fertility) when shifting from a 3-month to a 4-month DMPA dosing regimen.

## Name and Description of Product

The investigational product is Sayana® Press, consisting of 104 mg/0.65 mL MPA injectable suspension pre-filled in the Uniject^TM^ delivery device. The drug component of Sayana® Press, Depo-subQ Provera 104®, is approved by the US Food and Drug Administration (USFDA) as a contraceptive when delivered at a SC dose of 104 mg every 3 months (New Drug Application [NDA] # 21-583). The predominant method of action of the drug is the inhibition of gonadotropin-releasing hormone secretion, which suppresses levels of follicle stimulating hormone (FSH) and luteinizing hormone (LH), and in turn prevents follicular maturation and ovulation. Pfizer has not sought USFDA approval for the combination of drug and device which constitutes Sayana® Press, but it is registered in more than 36 countries and approved for self-injection in the United Kingdom [[17](#_ENREF_17)].

## Findings from Nonclinical and Clinical Studies

### Nonclinical Studies

The active pharmaceutical ingredient of Sayana® Press, MPA, has been marketed in various doses and formulations for decades. These include but are not limited to: 2.5, 5.0 and 10.0 mg oral tablets for the treatment of amenorrhea and uterine bleeding (NDA # 011839); a 150 mg/mL IM formulation for contraception (NDA # 20-246); and the 104 mg/0.65 mL SC formulation under study here, approved for contraception (NDA # 21-583) and the management of endometriosis-associated pain (NDA # 21-584).

The toxicology of MPA has been reviewed by the USFDA as part of recent marketing applications. Accordingly, a complete battery of toxicology studies has been conducted in compliance with the International Council for Harmonization (ICH) guidance for non-clinical safety studies. A thorough toxicology review was also performed by Jordan [[18](#_ENREF_18)]. Depo-Provera® CI did not show signs of chronic or reproductive toxicity, nor was there mutagenic or carcinogenic potential in animals. Multiple studies in different species have shown that DMPA has limited toxicity following administration by SC, IM or intravenous injection. The median lethal dose for intravenous administration was 376 mg/kg and 100 mg/kg in mice and rats, respectively. When given SC, the median lethal dose was increased to more than 4000 mg/kg and 8000 mg/kg in mice and rats, respectively [[18](#_ENREF_18)].

### Clinical Studies

#### Pharmacokinetics

The clinical pharmacology section of the Sayana® Press label was primarily informed by the study of Jain et al. which evaluated the PK profile of Depo-subQ Provera 104® and reported an average maximum serum concentration (C_max_), 3-month trough (C_3mo_), apparent terminal half-life (t_1/2_), and area under the serum concentration-time curve (AUC) extrapolated to infinity of 1.56ng/mL, 0.40 ng/mL, 43 days, and 92.8 ng•day/mL, respectively [[10](#_ENREF_10)]. Neither the manuscript nor the product label reports the site of injection used in the Jain study. Toh et al. evaluated the impact of injection site on PK of Depo-subQ Provera 104® among Asian women, and found a significantly lower C_max_ for injections in the abdomen than in the thigh (0.94 ng/mL versus 1.65 ng/mL). Mean t_1/2_ and C_3mo_ were larger for the abdomen (104 days and 0.47 ng/mL, respectively) than the thigh (81 days and 0.41 ng/mL), but the differences were not statistically significant [[19](#_ENREF_19)]. A PK/PD study comparing Sayana® Press to Depo-subQ Provera 104® in pre-filled glass syringe also failed to report the site of injection, but the average C_max_ for each device group (0.95 ng/mL and 0.79 ng/mL, respectively [[17](#_ENREF_17)]) was consistent with the abdominal data in Toh et al. [[19](#_ENREF_19)]. The mean MPA profile observed in a study of Depo-subQ Provera 104® injected in the upper arm was also more similar to the Toh et al. abdomen data [[20](#_ENREF_20)], although the variability of t_1/2_ and trough concentrations appeared higher with the upper arm injection site. Finally, the accumulation of MPA reported in an efficacy study of Depo-subQ Provera 104® [[12](#_ENREF_12)] was consistent with a noticeably longer t_1/2_ than reported by Jain et al. (Figure 1, inset). Overall, these data support the conclusion that, on average, the apparent terminal half-life of MPA may be longer than indicated by the Depo-subQ Provera 104® label, and that the PK of MPA may differ by injection site.


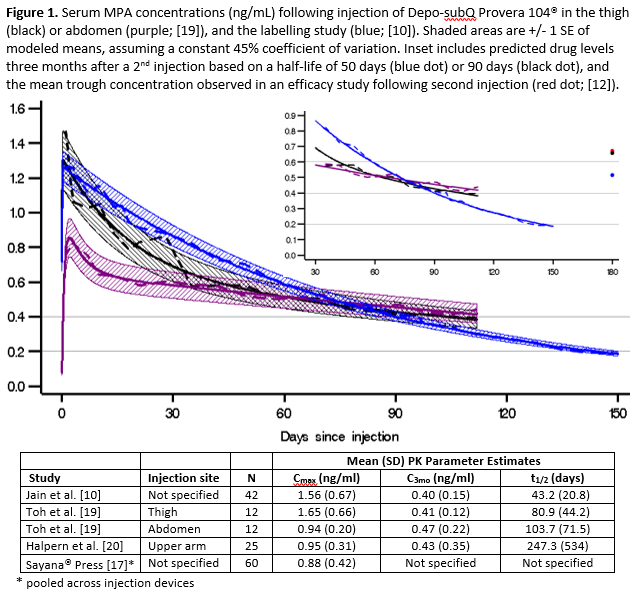


#### Pharmacodynamics

The study by Jain et al. [[10](#_ENREF_10)] is the only randomized trial comparing return to ovulation between Depo-subQ Provera 104® and DMPA-IM, and it was limited to 39 and 19 participants in the SC and IM groups, respectively. Other contemporaneous PD data for Depo-subQ Provera 104® include the study by Toh et al. [[19](#_ENREF_19)] and the Sayana® Press Public Assessment Report [[17](#_ENREF_17)]. Time to ovulation in these studies was based on a single elevated serum progesterone (P) concentration of 4.7 ng/mL or higher, supported with data on estradiol, LH, and FSH levels. The estimated cumulative probability of return to ovulation based on a meta-analysis of all the Depo-subQ Provera 104® data is presented in Figure 2. The timing of each ovulation was obtained by digitalizing data in the manuscript or using the event and censoring times specified in the reports (one event time that was only stated to have occurred before day 92 in the Sayana® Press study was imputed as day 46). Four participants with known dosing errors in the Sayana® Press study are excluded. All other PD outcomes are included, even if supportive data raised doubts in the minds of investigators as to whether they were true ovulations. Caution is required when making comparisons between groups due to the lack of randomization and the small number of DMPA-IM users. Nonetheless, the data are consistent with a significantly longer time to ovulation following initiation of Depo-subQ Provera 104® than DMPA-IM (p=0.02 based on a Wilcoxon log-rank statistic). Furthermore, only 1 of 96 women (1.0%) using Depo-subQ Provera 104® were observed to ovulate between days 90 and 120, compared to 2.4% who ovulated in the first 90 days using DMPA-IM.


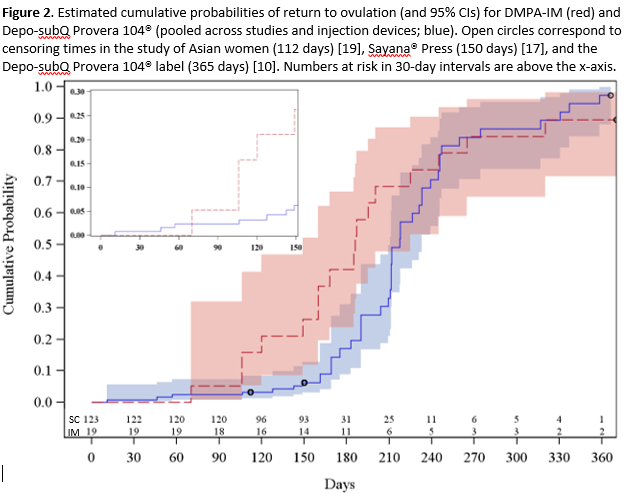


Overall, the combination of PK data which suggests an apparent longer terminal half-life of MPA than reported on the Depo-subQ Provera 104® label, and the PD data demonstrating longer return to ovulation among Depo-subQ Provera 104® users as compared to DMPA-IM users, suggests that the overall risk of pregnancy between months 3-4 post-dose may be low.

#### Clinical Efficacy Studies

There are no available data on the pregnancy rate for Depo-subQ Provera 104® when it is injected at intervals longer than three months (every 12-14 weeks). However, no pregnancies were detected among 2,042 women who used the product in the abdomen or thigh every 3 months for up to 3 years in three contraceptive studies that supported the NDA. When restricted to women aged 36 years or less at the time of treatment initiation, the 95% confidence interval for the Pearl Index (pregnancies per 100 WY of use) was 0.00-0.25 in the first year of use [[16](#_ENREF_16)].

## Known and Potential Risks and Benefits to Human Participants

### Known and Potential Health Risks of the Study Drug

#### Pregnancy

All women will be tested for pregnancy and the injection will be given during the first 5 days of the menstrual cycle to rule out pregnancy prior to enrollment. During the study, each woman will have a urine pregnancy test prior to receiving her second injection at month 4 and at month 12/study exit. A woman will also have a urine pregnancy test at month 8 if she is more than 7 days late for her third injection or enrolled in the BC-BMD sub-study, or any time during the study if she is experiencing any symptoms or signs of pregnancy or if she suspects she may be pregnant. Women participating in the extended follow-up subset will be tested for pregnancy at time points specified in Table 3. Based on the existing data the risk of pregnancy while using Sayana® Press injected every 4 months is assumed to be low (see Section 1.3.2). However, pregnancy rates will be monitored and if necessary, the pregnancy testing schedule may be modified and/or study participants informed if the actual pregnancy rate is higher than anticipated. Although no DMPA product should be used during pregnancy, there is little or no increased risk of birth defects in women who have inadvertently been exposed to MPA injections in early pregnancy [[7](#_ENREF_7)]. Neonates exposed to MPA in-utero and followed to adolescence showed no evidence of any adverse effects on their health including their physical, intellectual, sexual or social development [[21](#_ENREF_21)].

#### Injection Site Reactions

Clinical trials have demonstrated that there is a small risk of injection site reactions (ISRs) for Depo-subQ Provera 104®. In 5 clinical studies involving 2,325 women (282 treated for up to two injection intervals; 1,780 treated for up to four injection intervals; and 263 women treated for up to 8 injection intervals), 5% reported ISRs and 1% had persistent skin changes, typically described as small areas of induration or atrophy [[12](#_ENREF_12), [16](#_ENREF_16)]. A retrospective study based on individual case safety reports of women receiving Depo-subQ Provera 104® identified 398 injection site reactions reported to the WHO VigiBase through January 2016. Notably, the rate of ISRs in the retrospective study could not be quantified due to the unknown total number of women and injections [[22](#_ENREF_22)].

#### Menstrual Changes

Most women using Depo-subQ Provera 104® experience disruption of menstrual bleeding patterns. Altered menstrual bleeding patterns include amenorrhea, irregular or unpredictable bleeding or spotting, prolonged spotting or bleeding, and heavy bleeding. As women continue using Depo-subQ Provera 104®, fewer experience irregular bleeding and more experience amenorrhea. In three efficacy trials, the rates of amenorrhea were 39% at month 6 and 56.5% at month 12 [[16](#_ENREF_16)]. It is unknown whether extending the dosing interval to 4 months will alter these patterns.

#### Weight Gain

Women tend to gain weight when using Depo-subQ Provera 104® every 3 months, with an average increase of 1.6 kg observed after 1 year. Longer term effects of Depo-subQ Provera 104® are unknown; however, women using DMPA-IM gain an average of 6.6 kg after 10 years of uninterrupted use [[16](#_ENREF_16), [23](#_ENREF_23)]. We will assess the weight of participants during the study to characterize the pattern of weight change when dosing every 4 months. In addition, the Brazil site will measure changes in body composition (BC) – including but not limited to total body mass, lean and fat mass, percentage of total fat mass, and central-peripheral fat ratio – in a subset of Brazilian users of Sayana® Press at baseline, month 8 and month 12.

#### Risk of HIV

Depo-subQ Provera 104® provides no protection against sexually transmitted infections (STIs), including HIV. While some observational data have suggested an association between the use of DMPA-IM and an increased risk of HIV acquisition [[8](#_ENREF_8), [9](#_ENREF_9)], the data are inconsistent [[9](#_ENREF_9), [24](#_ENREF_24), [25](#_ENREF_25)]. In light of the inconclusive evidence, the WHO recently revised their Medical Eligibility Criteria for contraceptive use [[26](#_ENREF_26)]. Per the revised criteria, women at low risk of HIV can continue using all hormonal methods of contraception including DMPA without restriction. Women who are at high risk of HIV, however, can use DMPA if they are informed of the possible increased risk of HIV acquisition among DMPA users, the uncertainty over a causal relationship, and how to minimize their risk of acquiring HIV. Notably, no studies have assessed the relationship between the lower-dose Depo-subQ Provera 104® product and HIV. The WHO and US Centers for Disease Control recommend that women at high risk of HIV who choose to use DMPA or any other hormonal product should be counseled to use condoms consistently and correctly [[25](#_ENREF_25)]. This study will not recruit women at known risk of HIV, including women with partners who are known to be HIV-positive at baseline. However, women will be provided the information required by WHO in the informed consent and will be counseled to use condoms for protection against HIV/STI if their risk status changes during the study.

#### Return to fertility

Return to fertility is delayed after stopping the use of Depo-subQ Provera 104® when injected every 3 months. Among 28 women in clinical studies who stopped treatment to become pregnant, only 1 became pregnant within 1 year of her last injection. Similarly, among 15 women who received multiple doses of Depo-subQ Provera 104®, the median time to ovulation was 10 months after the last injection, and only 12 women (80%) ovulated within 1 year [[16](#_ENREF_16)]. Extending the injection interval from 3 to 4 months is anticipated to reduce the accumulation of MPA concentrations and hence the delay in return to ovulation and fertility. Nevertheless, only women who do not desire pregnancy for a minimum of 18 months (10 months from last study injection) will be enrolled in the study.

#### Bone Mineral Density

A boxed warning regarding skeletal health was added to the label of Depo-subQ Provera 104® in 2004 indicating that long-term use (more than 2 years) may reduce peak bone mass and place women at increased risk for osteoporotic fractures [[16](#_ENREF_16)]. Changes in BMD are reversible and comparable to the changes associated with pregnancy and lactation. Also, no clinical evidence exists that long-term use of DMPA is associated with increased risk of fracture [[27](#_ENREF_27)]. After reviewing the evidence, WHO concluded that there should be no restriction on the use of the drug among women aged 18 to 45, including no restriction on duration of use [[28](#_ENREF_28)]. The Brazil site will assess BMD at the lumbar spine (LS) (L1-L4) and femoral neck (FN) in a subset of Brazilian users of Sayana® Press at baseline, month 8 and month 12.

#### Other Medical Risks

Additional information regarding possible risks of Depo-subQ Provera 104® use may be found in the package insert [[16](#_ENREF_16)] and the Sayana® Press package leaflet [[29](#_ENREF_29)].

#### Loss of Confidentiality

All study staff will protect the confidentiality of participants to the fullest extent possible, except as required by law. No participant personal identifiers will appear on any data or documentation sent to FHI 360. Participants will not be identified by name in any report or publication resulting from study data. Loss of confidentiality may present a risk to study participants if these procedures are inadvertently breached.

### Benefits to Participants

There will be no direct benefits of study participation.

## Selection of Drugs and Dosages

Sayana® Press (104 mg MPA in 0.65 mL injectable suspension) is the only drug that will be used in this study. Injections will be given subcutaneously in the abdomen, upper thigh or back of the upper arm every 4 months for a 12-month treatment period (3 doses). The drug, dosage and regimen were chosen based on the objectives of the trial: to assess the effectiveness, PK, safety, and acceptability of Sayana® Press when injected every 4 months.

## Compliance Statement

Before implementation, the protocol and relevant study documents including recruitment materials will be approved by the study sites’ local institutional review boards (IRBs) and FHI 360’s Protection of Human Subjects Committee (PHSC). The study will be conducted in compliance with FHI 360 Standard Operating Procedures (SOPs), and in accordance with ICH Good Clinical Practice (GCP) Consolidated Guideline (E6) and any other applicable national and local laws and regulations. Any episode of noncompliance with these guidelines and regulations will be documented in the Trial Master File and reported to national regulatory authorities and governing IRBs as required.

Site investigators are responsible for performing the study at his or her site in accordance with the protocol, applicable GCP guidelines, and IRB and local regulatory authority requirements. Agreement of each site investigator to conduct the study in accordance with the protocol will be documented on the Investigator Signature form, in separate study agreements with FHI 360, and other applicable forms as required by national authorities. The site investigators will permit trial-related monitoring, audits, ethical review and regulatory inspections by providing direct access to source data and other study-related documents.

Site investigators are also responsible for ensuring the privacy, health, and welfare of participants during the study, and will ensure that trained personnel are immediately available in the event of a medical emergency. Site investigators and applicable study staff will be trained in research ethics prior to working on the study and must be familiar with the properties and side effects of Depo-subQ Provera 104® and Sayana® Press as described in the prescribing information.

## Population to Be Studied

Healthy, sexually active female participants between 18 and 35 years of age at the time of enrollment who are willing to use Sayana® Press injected every 4 months as the only means of contraception for 12 months.

# PURPOSE OF THE STUDY AND STUDY OBJECTIVES

## Purpose of the Study

The purpose of the study is to evaluate the effectiveness, PK, safety, and acceptability of Sayana® Press when injected every 4 months (17-18 weeks) rather than the currently prescribed 3-month (12-14 weeks) regimen.

## Study Objectives

### Primary Objective

To evaluate the effectiveness of Sayana® Press when injected subcutaneously every 4 months in the abdomen or upper thigh for 12 months (3 treatment cycles) of use.

### Secondary Objectives

The secondary objectives of the study are to:

- Assess trough concentrations, accumulation, and apparent terminal half-life of MPA, and the impact of subcutaneous injection site (abdomen, upper thigh, or back of the upper arm) on these parameters, when Sayana® Press is injected every 4 months for 12 months of use
- Evaluate the safety of Sayana® Press when injected every 4 months for 12 months of use
- Evaluate the acceptability of Sayana® Press when injected every 4 months for 12 months of use

### Exploratory Objective

To evaluate return to ovulation among a subset of study participants who have received both month 4 and month 8 injections and plan to use non-hormonal methods of contraception, or no contraception, for up to 12 months from the last study injection.

## Study Endpoints

### Primary Endpoint

The primary study endpoint is the occurrence of pregnancy, as defined by a positive urine pregnancy test. Positive urine tests will be confirmed by ultrasound and/or serum hCG testing whenever possible.

### Secondary Endpoint

Secondary endpoints include serum MPA concentrations on day 0 (baseline) and months 2, 3, 4, 8, and 12 in a subset of 120 participants; serious adverse events (SAEs) and adverse events (AEs) leading to product withdrawal; ISRs, bleeding patterns, blood pressure (BP) and body weight; and responses to acceptability questions.

### Exploratory Endpoint

Return to ovulation, where ovulation is defined as a single elevated serum progesterone (P ≥4.7 ng/mL). Given that ovulation must precede conception, for the purpose of this analysis, a confirmed pregnancy test (regardless of progesterone results) will be considered an ovulation.

# STUDY DESIGN

## General Design and Study Schema

This study will evaluate the effectiveness, PK, safety, and acceptability of Sayana® Press when injected every 4 months (17-18 weeks) for 12 months (3 treatment cycles), rather than every 3 months (12-14 weeks) per current prescribing information. A total of 750 healthy, sexually active women aged 18 to 35 years with regular menstrual cycles and at risk of pregnancy will be enrolled at 3 sites: Biomedical Research Department at Profamilia in the Dominican Republic (Profamilia); Universidade Estadual de Campinas in Brazil (UNICAMP), and Instituto Chileno de Medicina Reproductiva in Chile (ICMER). One cohort of N=630 women will be randomized 1:1 to receive injections of Sayana® Press in the abdomen or upper thigh, and a second PK cohort of N=120 women who, in addition to pregnancy testing, agree to blood draws for MPA testing at 6 time points during their study participation, will be randomized 1:1:1 to receive injections in the abdomen, upper thigh, or back of the upper arm. The N=710 women receiving injections in the abdomen or thigh, per Sayana® Press package insert instructions, will constitute the primary population for assessing contraceptive efficacy. However, because upper arm injections are supported by PATH instructional materials, and have been reported as the preferred injection site in prior studies, analyses will be done to compare PK across the three injection sites [[14](#_ENREF_14)].

Treatment will begin in the first 5 days of menses. All participants will have a single serum sample collected at enrollment for possible MPA testing. Participants will receive re-injections in their randomly assigned SC injection site at month 4 (17 weeks after treatment initiation) and at month 8 (35 weeks after treatment initiation), and complete their scheduled follow-up at month 12 (53 weeks after treatment initiation). There will be a plus 7-day grace period for re-injections, but women who are up to 28 days late may continue treatment if they have a negative urine pregnancy test (participants who are more than 28 days late for re-injection will be discontinued from the study). Participants will be asked to provide information on adverse events, prohibited concomitant medication and other contraceptive use, vaginal bleeding, and method acceptability at months 4, 8, and 12; information on adverse events and concomitant medications will be recorded if self-reported by participants at the month 2 and 3 PK visits, and any unscheduled visits. Urine pregnancy testing will be performed for all women at month 4 and month 12/study exit, if a participant is more than 7 days late for her month 8 visit and at any other time it is clinically indicated. An additional pregnancy test will be completed at month 8 for women enrolled in BC-BMD sub-study in Brazil. Participants will be advised to return to the clinic for unscheduled visits any time they think they may be pregnant or have a medical problem that may be related to their study participation. In addition to pregnancy testing, participants in the PK cohort will provide blood specimens for serum MPA testing on day 0 (baseline) and months 2, 3, 4, 8, and 12.

A subset of approximately 20 participants at select study sites who received both month 4 and month 8 injections and plan to use a non-hormonal contraceptive method, or no contraception, after study discontinuation will be invited to be in the extended follow-up subset and monitored until resumption of ovulation or up to a maximum of 12 months after the last study injection, whichever comes first. At predetermined time points (specified in Table 2 and Table 3), blood will be collected for progesterone, estradiol, MPA and other synthetic steroids (e.g., levonorgestrel, etonorgestrel and ethinyl estradiol); urine pregnancy testing will be performed; and blood pressure and weight will be measured.

DMPA-IM has been associated with a possible increased risk of HIV acquisition and an ongoing randomized controlled trial (ECHO) will provide the first prospective data comparing risk with DMPA-IM compared to other contraceptive methods. There are no data available to assess the relationship between the lower subcutaneous administered dose of Depo-SubQ Provera 104® (104 mg MPA) and HIV acquisition [[24](#_ENREF_24)]. The putative biologic mechanisms that might underlie a potential association between DMPA and HIV are poorly understood, but are believed to be related to the effects of DMPA on the immune environment in the female genital tract. One of the hypotheses concerning the mechanism of this potential association is the hypoestrogenic effects of DMPA. Another is a direct effect of MPA on the glucocorticoid receptor, leading to immunosuppression. In in vitro studies, the effects of MPA have been shown to vary with concentration, with some markers showing dose-response effects [[30](#_ENREF_30)]. However, no clinical studies have evaluated the effects of different DMPA dosages and serum hormone concentrations on female genital tract immunity in vivo. FHI 360 is planning a study to evaluate the effect of different dosages of DMPA on soluble and cellular markers of genital tract immunity and on the genital microbiome (referred to here and thereafter as the Vaginal Immunity Study). The outcomes of that study should provide comparative information on markers of vaginal immunity and assess whether the lower doses of DMPA are potentially safer for women in terms of acquisition of HIV and other sexually transmitted infections (STIs). At select sites, we will ask participants in this study if they are willing to provide a blood sample and vaginal and cervical swabs for the Vaginal Immunity Study. Participants who agree to collection of additional specimens for the Vaginal Immunity Study will provide blood and vaginal specimens at the following visits: screening and follow-up months 1, 3, 4, 8 and 12/final.

There is scarce information about BC and BMD among users of Depo-subQ Provera 104®. The Brazil site plans to conduct the BC-BMD sub-study to assess BC and bone health in Brazilian users of Sayana® Press when injected every 4 months for 12 months of use. At that site only, we will ask participants in this study if they are willing to provide additional BC and BMD measurements. A sub-study cohort of N=139 will be achieved by enrolling the first 139 women agreeing to participate. Participants who consent will undergo Dual Energy X-ray absorptiometry (DXA) imaging (GE Healthcare, Lunar Corporation, Madison, Wisconsin, USA) at day 0 (baseline), month 8 and month 12 after treatment initiation. At month 8, urine pregnancy testing will be performed for BC-BMD participants prior to radiation exposure. The site will be responsible for all activities related to this sub-study, with the exception of CRF entry.

## Justification for the Study Design

The study is designed to meet criteria for assessing the efficacy of hormonal contraceptive products specified in the European Medicines Agency Guideline on Clinical Investigation of Steroid Contraceptives in Women[[31](#_ENREF_31)]. Specifically, at least 400 women completing one year of use and sufficient total months of use to obtain a 95% confidence interval for the Pearl Index (pregnancies per 100 WY of follow-up) with a half-width of no more than 1%. A study size of 710 women receiving injections in the abdomen or thigh is expected to achieve these criteria, while also allowing us to assess whether pregnancy risk may differ between these two injection sites.

The PK cohort will allow us to inform an appropriate grace period for re-injections based on estimated trough concentrations and apparent terminal half-life. Although the Product Leaflet for Sayana® Press specifies that injections are to be administered in the abdomen or thigh [[29](#_ENREF_29)], back of the upper arm injection instructions are provided by PATH and the back of the upper arm is used by some providers ([[32](#_ENREF_32)], Appendix 1). By randomizing an additional N=40 women to receive injections in the back of the upper arm we will be able to confirm findings from a previous non-comparative PK study which indicated MPA concentrations are more variable when drug is administered in this anatomical site [[20](#_ENREF_20)], and inform the potential impact of any excess variability on efficacy.

To inform how much sooner women may expect to return to ovulation (and therefore, to fertility) when shifting from a 3-month to a 4-month dosing regimen, we will evaluate return to ovulation among a subset of study participants who received both month 4 and month 8 injections and plan to use a non-hormonal method of contraception, or no contraception, after study discontinuation, until resumption of ovulation or up to a maximum of 12 months from the last study injection, whichever comes first. We will also test for pregnancy, and measure levels of MPA and other synthetic steroids (e.g., levonorgestrel, etonogestrel and ethinyl estradiol) to rule out concurrent use of other hormonal contraceptives that may suppress ovarian activity.

## Efficacy Measure

The primary efficacy measure is the pregnancy Pearl Index, computed as the number of pregnancies occurring during the treatment per 100 WY of follow-up. Time to pregnancy will be based on the difference between the date of treatment initiation and the estimated date of fertilization (EDF), where the latter is determined by ultrasound, the first day of last menstrual period (LMP) prior to the positive pregnancy test, or using best clinical judgment in the absence of these results. Participants who do not become pregnant will be censored on the date of their last negative urine pregnancy test or the end of their treatment use period, whichever is earlier (see Section 10.6 for definition of treatment use period).

## Pharmacokinetic Measures

The PK of MPA will be evaluated among participants in the PK cohort based on estimated trough concentrations at month 4 (C_4mo_), month 8 (C_8mo_), and month 12 (C_12mo_); apparent half-life (t_1/2_); and accumulation of MPA over 3 cycles of use.

## Safety Measures

Safety measures will include the occurrence of SAEs, AEs leading to product withdrawal, ISRs, change in bleeding pattern, change from baseline in BP and body weight at the month 4, 8, and final visits.

## Acceptability Measures

Method acceptability will be assessed based on responses to questions about vaginal bleeding (at baseline) and other side effects, likes, and dislikes about the regimen at month 4, 8, and final visits.

## Randomization, Allocation Concealment and Blinding

### Randomization

All participants will receive the same study drug, Sayana® Press. However, women will be randomized to receive their injections in the abdomen, back of the upper arm, or upper thigh to control for potential confounding when assessing relationships between SC injection site, efficacy, and PK. One cohort of N=630 women will be randomized 1:1 to receive their injections in the abdomen or thigh, and a separate PK cohort of N=120 women agreeing to MPA testing will be randomized 1:1:1 to receive their injections in the abdomen, upper thigh, or back of the upper arm. Randomization will be stratified by cohort and site using appropriate randomly permuted block sizes. Randomization sequences will be created for each combination of site and cohort by an FHI 360 randomization statistician using a validated program written in SAS® version 9.4 or higher.

### Allocation Concealment

Sequentially numbered, opaque randomization envelopes matching the randomization sequences will be provided to each clinic site. Each envelope will have the randomization number printed on the outside, with the corresponding SC injection site assignment concealed within. The randomization envelopes will be maintained in a secure office, with access limited to designated study staff. The next available randomization number will be assigned to the next study participant only after she has been confirmed to be eligible, consented to participate, and undergone all other enrollment procedures, including having a blood sample collected for MPA testing among women agreeing to participate in the PK cohort.

### Blinding

Neither participants nor site staff will be blinded to SC injection sites. However, laboratory staff analyzing serum MPA specimens and staff at FHI 360 responsible for adjudicating pregnancy outcomes and coding adverse events will be blinded to individual participant injection site assignments for the duration of each participant’s follow up. In addition, the FHI 360 may review interim PK data, including MPA concentrations of any women who become pregnant, to inform other FHI 360 MPA projects.

## Data and Safety Monitoring Board

An independent Data and Safety Monitoring Board (DSMB) will review interim data when 250 participants have completed 4 months of follow-up. This single, pre-planned interim analysis is intended to inform a decision whether to stop or modify the study if the estimated probability of pregnancy is greater than 2% in the first injection interval. Additional interim analyses may be requested by FHI 360 or the DSMB based on periodic reviews of study indicators (pregnancy, AE, recruitment and continuation rates) if deemed necessary to protect participant safety or to make recommendations to modify the study.

## Study Drug and Dosage

Sayana® Press (MPA injectable suspension, 104 mg/0.65 mL, pre-filled in the Uniject^TM^ delivery injection system) is indicated for the prevention of pregnancy. The composition of each 0.65 mL dose of drug is shown in Table 1. Sayana® Press will be administered as a SC injection every 4 months, rather than every 3 months per current prescribing information. Instructional materials developed by PATH, which are consistent with the prescribing information, will be followed when providing injections [[32](#_ENREF_32)]; Appendix 1).

**Table 1. Composition of Depo-subQ Provera 104®**

| Ingredients | Amount per 0.65mL |
| --- | --- |
| Active: |  |
| MPA | 104 mg |
| Inactive: |  |
| Polysorbate 80 | 1.95 mg |
| Sodium chloride | 5.2 mg |
| Methionine-Phosphate buffer | 17.47 mM |
| Povidone | 3.25 mg |
| Methylparaben | 1.04 mg |
| Propylparaben | 0.098 mg |
| Water for injection | qs to 0.65 mL |

## Drug Supply and Accountability

### Drug Accountability

The site investigator or designee will acknowledge receipt of study product indicating shipment content and condition, and follow drug accountability procedures throughout the study. Drug accountability includes maintaining accurate records on the quantity of product received, date of receipt, condition at receipt, lot number of products received and dispensed, description of damaged units, dispensation, and product disposition or destruction. Storage temperature continuity tracking will occur during storage. Site personnel are responsible for routine temperature monitoring and safe storage of the study products after they are received on-site. Study drug product will only be administered to participants enrolled in the trial; unused units will be disposed of per FHI 360 written instructions.

## Duration of Study Participation

This study will consist of a screening period (up to 1 month prior to enrollment) and a 12-month treatment period, for approximately 13 months of study participation per subject. A subset of approximately 20 participants will be followed until the resumption of ovulation or a maximum of 12 months from the last study injection, whichever comes first.

## Discontinuation Criteria

### Study Termination

This study may be discontinued at any time by FHI 360, FHI 360’s PHSC, applicable government or regulatory authorities, or site IRBs. If the study is closed early for any reason, site investigators and IRBs will be promptly notified. Site investigators will inform study participants and assure that final visit procedures are completed whenever possible.

### Participant Discontinuation

A participant may discontinue participation at any time for any reason without loss of other benefits or services to which she may be entitled. A participant may also be discontinued early from the study if the site investigator considers it to be in the participant's best interest. Final visit procedures should be completed for all participants who discontinue early, if possible. Reasons for discontinuation from the study will be recorded on the appropriate Case Report Form (CRF).

### Drug Supply, Storage and Security

Sayana® Press is manufactured and distributed by Pfizer, Inc. Drug supply will be procured by USAID for this study. If applicable, prior to initiation, the product will be labeled per local regulatory agency requirements. Drug product will be stored in a secure, dry place at controlled room temperature of 15 to 30°C according to the manufacturer’s stipulations.

## Source Data Recorded on Case Report Forms

Some CRF fields will have data directly recorded into them and will be considered source. Other source documents will include, but will not be limited to, clinical notes, laboratory results, dispensing records and consent forms. Source for all study data will be defined and documented in site-specific source documentation guides.

MPA testing will be conducted by PPD Development (Richmond, VA, USA), which will maintain source documentation for MPA data. MPA results will not be entered into CRFs but transferred electronically to the FHI 360 in accordance with written study-specific procedures.

## Study Procedures

All participants will come to the study clinic for a screening visit, an enrollment visit (when treatment initiation occurs), re-injection visits at month 4 and month 8 post-treatment initiation, and a final visit at month 12. Participants in the PK cohort will attend additional visits at months 2 and 3 to provide blood specimens for MPA testing. Participants in the extended follow-up subset will return at predetermined time points (specified in Table 3) until return to ovulation or up to a maximum of 12 months from the last study injection, whichever comes first. See Table 2 and Table 3 for timing of study procedures.

**Table 2: Study Procedures and Assessments**

| **Procedures** | **Screening ^a^** | **Enrollment (Day 0)** | **Month 1** | **Months**  **2 and 3 ^b^** | **Months 4 and 8** | **Final/**  **Month 12 ^k^** |
| --- | --- | --- | --- | --- | --- | --- |
| Informed consent | X |  |  |  |  | X ^l^ |
| Inclusion/exclusion criteria | X | X |  |  |  |  |
| Baseline characteristics | X | X |  |  |  |  |
| Height |  | X |  |  |  |  |
| Body weight |  | X |  | X | X | X |
| Blood pressure |  | X |  | X | X | X |
| Urine pregnancy test | X ^c^ | X |  |  | X ^d^ | X |
| Randomization |  | X |  |  |  |  |
| Drug administration |  | X |  |  | X |  |
| Serum MPA |  | X |  | X^e^ | X^e^ | X^e,l^ |
| AE documentation ^f^ |  |  |  | X | X | X |
| ISR documentation |  | X |  |  | X | X |
| Concomitant medication documentation ^g^ |  |  |  | X | X | X |
| Vaginal bleeding |  | X |  |  | X | X |
| Acceptability |  |  |  |  | X | X |
| Coital frequency and condom use |  | X |  |  | X | X |
| Specimens for vaginal immunity study ^h^ | X |  | X | X | X | X |
| DXA for BC and BMD sub-study ^i^ |  | X |  |  | X ^j^ | X |

^a^ May coincide with enrollment visit if participant is in the first 5 days of her menses (this does not apply to women also participating in the Vaginal Immunity Study)

^b^ Only for the PK cohort

^c^ If clinically indicated

^d^ Participants will only be tested at month 8 if they are more than 7 days late for their visit or are enrolled in the BC-BMD sub-study

^e^ For all participants at enrollment. At follow-up visits only for the PK cohort and for any participant in the full cohort who has a positive pregnancy test during follow-up

^f^ Serious adverse events, adverse events leading to product withdrawal, and injection site reactions that are Grade 1 or higher will be recorded on the AE CRF

^g^ Use of prohibited concomitant medications and other contraceptives, will be recorded on CRFs

^h^ Participants who consent to the collection of genital and blood specimens for the Vaginal Immunity Study will also be asked to come to the clinic approximately 1 and 3 months after enrollment for specimen collection only.

^i^ Only for Brazilian participants who consent to measurement of BC and BMD by DXA scan. Does not need to be done in the case of early discontinuation.

^j^ Only at month 8 for BC-BMD participants.

^k^ Procedures to be done at the Month 12 visit, or at any visit of early discontinuation.

^l^ Only for the extended follow-up subset; may occur after the Final/Month 12 visit so long as the participant meets the eligibility criteria for the extended cohort and the timetable for extended follow-up procedures.

**Table 3: Study Procedures and Assessments (Extended Follow-Up Subset)**

| **Procedures ^a^** | **Week 38** ^d^ | **Week 39** ^d^ | **Week 40** ^d^ | **Week 41** ^d^ | **Week 42** ^d^ | **Week 46** ^d^ | **Week 47** ^d^ | **Week 48** ^d^ | **Week 49** ^d^ | **Week 50** ^d^ |
| --- | --- | --- | --- | --- | --- | --- | --- | --- | --- | --- |
| Body weight | X |  |  |  |  | X |  |  |  |  |
| Blood pressure | X |  |  |  |  | X |  |  |  |  |
| Urine pregnancy test ^b^ | X | X ^c^ | X ^c^ | X ^c^ | X | X | X ^c^ | X ^c^ | X ^c^ | X |
| Serum MPA | X |  |  |  |  | X |  |  |  |  |
| Serum Estradiol | X | X | X | X | X | X | X | X | X | X |
| Serum Progesterone | X | X | X | X | X | X | X | X | X | X |
| Plasma levonorgestrel, etonogestrel, ethinyl estradiol | X |  |  |  |  | X |  |  |  |  |
| AE documentation ^e^ |  |  |  |  |  |  |  |  |  |  |
| Concomitant medication documentation ^f^ |  |  |  |  |  |  |  |  |  |  |

^a^ Participants will be discontinued from the study at the visit in which ovulation is confirmed (defined as a single elevated serum progesterone [P ≥4.7 ng/mL] or confirmed pregnancy test) or she does not ovulate by week 50 (from last injection), whichever occurs first

^b^ If positive at any time, discontinue

^c^ If clinically indicated

^d^ Weeks from last injection

^e^ Only SAEs will be recorded on study CRFs and reported to FHI 360

^f^ Use of prohibited concomitant medications and other contraceptives, will be recorded on CRFs

### Screening Visit

Women will be recruited through various channels which may include, but not be limited to, searches of internal participant databases, informing women seeking contraception at the study sites about the study, referrals from local clinics and use of advertisements. Women expressing interest in participating in the study will be given the informed consent form to review and trained site personnel will review the consent information including the study objectives, design and procedures, and potential risks of participation. Potentially eligible women will be asked if they are willing to be in the PK cohort, and provide blood specimens for MPA testing, until enrollment into the PK cohort is complete. Those that sign the informed consent form will be assigned a participant number and have a screening interview, including collection of medical history and demographic information. Each site may also conduct clinical procedures per site-specific standard of care that are not required per the study protocol (e.g., STI testing, Pap smear, and breast exam).

Each potentially eligible woman will be scheduled to return to clinic for enrollment procedures during the first 5 days of her next menses, but if she is currently in the first 5 days of menses then enrollment procedures can begin immediately (this does not apply to women who also consent to participate in the Vaginal Immunity Study). In summary, the following will be done during the screening visit:

• Study purpose and procedures explained

• Written informed consent obtained

• Participant number assigned

• Assess study eligibility and collect baseline data

• Enrollment visit scheduled for first 5 days of next menses

• Appropriate CRFs completed

### Enrollment Visit (Day 0)

The enrollment visit will take place during the first 5 days of menses. During this visit a urine pregnancy test will be performed and other eligibility criteria verified. Height, weight and BP measurements will be recorded. Vaginal bleeding information will be collected. Approximately 5 mL of blood for MPA testing will be collected before randomization.

After a participant is confirmed eligible and had all other enrollment procedures completed, the study site’s randomization manager will open the next sequentially numbered randomization envelope from the appropriate stream of envelopes (PK cohort or non-PK cohort). Each participant will receive an injection of Sayana® Press in her assigned SC injection site, be monitored for at least 15 minutes for possible anaphylactic reactions and/or ISRs, and be scheduled to return to the clinic 4 months later for re-injection on study day 119 (17 weeks after enrollment) (participants who are unable to return precisely on study day 119 may have their re-injection scheduled to take place up to 7-days late, on or before study day 126). Participants in the PK cohort will be scheduled to return for additional blood draw visits at month 2 (study day 61) and month 3 (study day 91), allowing for a plus/minus 7-day grace period.

In summary, the following will be done during the enrollment visit:

- Urine pregnancy test performed
- Study eligibility confirmed and baseline data collected
- Coital frequency and condom use assessed
- Vaginal bleeding assessed
- BP, height, and weight measured
- Blood drawn for MPA assessment (MPA testing will only be done on specimens from participants in the main cohort if MPA is detected above the threshold defined in the Statistical Analysis Plan among baseline specimens from the PK cohort)
- Randomization completed
- Study drug administered and site of injection evaluated for possible ISRs
- Study visit schedule provided
- Appropriate CRFs completed

### Regularly Scheduled Follow-up Visits

#### Month 4 re-injection

The first re-injection visit is scheduled to occur on study day 119, 4 months (17 weeks) after treatment initiation. Participants will be asked if they have experienced any medical problems or have taken any medications since the previous visit (serious adverse events, adverse events leading to product withdrawal, ISRs and use of any study prohibited concomitant medications and other contraceptives , will be recorded on CRFs); asked to report on coital frequency and condom use since the last injection of study drug; have vaginal bleeding and product use acceptability evaluated; have BP and weight measured; and complete a urine pregnancy test. Any participants who are determined to be pregnant will have a blood specimen collected for MPA testing. Participants in the PK cohort will also provide approximately 5 mL of blood for MPA testing. Otherwise, participants who have a negative urine pregnancy test will receive a re-injection of Sayana® Press and be reminded to return for the next scheduled visit on study day 245, 8 months (35 weeks) after treatment initiation.

Participants who are up to 28 days late for the month 4 visit may continue treatment if they have a negative urine pregnancy test result. If a participant is more than 7 days late, however, then her month 8 and final study visits will be re-scheduled to occur at 18-week (126 day) intervals over the next 36 weeks in order to avoid shortening the effective lengths of subsequent treatment intervals. Participants who are more than 28 days late will undergo final visit procedures (see Section 3.14.3.3) and be discontinued from the study.

#### Month 8 re-injection

The second re-injection visit is schedule to occur on study day 245, 8 months (35 weeks) after treatment initiation. Participants will be asked if they have experienced any medical problems or have taken any medications since the previous visit (serious adverse events and adverse events leading to product withdrawal, ISRs, and use of any study prohibited concomitant medications and other contraceptives , will be recorded on CRFs); asked to report on coital frequency and condom use since the last injection of study drug; have vaginal bleeding and product use acceptability evaluated; and have BP and weight measured. A urine pregnancy test will not be performed unless the participant is more than 7 days late for her month 8 visit, she is enrolled in the BC-BMD sub-study, or if testing is clinically indicated. Any participants who are determined to be pregnant will have a blood specimen collected for MPA testing. Participants in the PK cohort will also provide approximately 5 mL of blood for MPA testing. Otherwise, participants who are not pregnant will receive a re-injection of Sayana® Press and be reminded to return for a final visit on study day 371, 12 months (53 weeks) after treatment initiation.

Participants who are 8 to 28 days late for the month 8 visit may continue treatment if the urine pregnancy test result is negative. If a participant is more than 7 days late, however, then the final study visit will be re-scheduled to occur 18 weeks (126 days) later. Participants who are more than 28 days late will undergo final visit procedures (see Section 3.14.3.3) and be discontinued from the study.

#### Final visit

The final visit is scheduled to occur on study day 371, 12 months (53 weeks) after treatment initiation. Participants will be asked if they have experienced any medical problems or have taken any medications since the previous visit; asked to report on coital frequency and any condom use since the last injection of study drug; have final vaginal bleeding and product use acceptability evaluated; have BP and weight measured; and complete a urine pregnancy test. Any participants who are determined to be pregnant will have a blood specimen collected for MPA testing. Interested participants who meet qualifications will be consented to the extended follow-up subset. Participants in the PK cohort and extended follow-up subset will also provide approximately 5 mL of a blood for MPA testing. Participants will be offered contraception of their choice from methods available at the study clinic and will be provided referrals as need for ongoing services. Final visit procedures should be done at the time of any early discontinuation.

In summary, the following will be done during regularly scheduled re-injection and final study visits:

- Participant asked about adverse events and concomitant medication use (serious adverse events, adverse events leading to product withdrawal, ISRs, and use of any study prohibited concomitant medications and other contraceptives, will be recorded on CRFs)
- Coital frequency and condom use assessed
- Vaginal bleeding and product use acceptability assessed
- BP and weight measured
- Consented to the extended follow-up subset*
- Blood drawn for MPA assessment (PK cohort, extended follow-up subset*, or if pregnant, only)
- Urine pregnancy test performed (at month 8 visit, only if more than 7 days late, or enrolled in the BC-BMD sub-study)
- Study drug administered (month 4 and month 8, only; contraception [locally available] offered at final visit)
- Site of the previous and new injection(s) evaluated for possible ISRs, as appropriate
- Appointment reminders provided (month 4, month 8, and extended follow-up subset only)
- Appropriate CRFs completed

*May occur after the Final/Month 12 visit so long as the participant meets the eligibility criteria for the extended cohort and the timetable for extended follow-up procedures.

### Additional Blood Draw Visits (Months 2 and 3)

Participants in the PK cohort will complete additional clinic visits to provide blood specimens for MPA testing at month 2 (study day 61) and month 3 (day 91). Blood pressure and weight will be measured; information on AEs and concomitant medication use will not be solicited; however, if reported by the participant, serious adverse events and adverse events leading to product withdrawal, and use of any study prohibited concomitant medications and other contraceptives, will be recorded on CRFs. Additional blood draw visits will be scheduled to allow for a plus or minus 7-day grace period, but women returning outside the window will still have blood drawn for MPA testing.

### Additional Vaginal, Endocervical and Blood Samples

At select study sites, consenting participants will provide additional vaginal, endocervical and blood specimens for the Vaginal Immunity Study at the following timepoints: screening and follow-up months 1, 3, 4, 8 and 12/final. Commercially available swabs and cytobrushes will be procured by the study sites, or if needed, provided by FHI 360. The collected specimens will be stored at -80°C until further shipment instructions from FHI 360. Additional instructions for the collection, processing and storage of specimens will be standardized in the study manual.

### Additional BC and BMD Measurements

At the Brazil site only, consenting participants will undergo DXA imaging for the BC-BMD sub-study at enrollment, month 8 and month 12. BC measurements will include, but are not limited to, total body mass, lean and fat mass, percentage of total fat mass, central-peripheral fat ratio, and Fat Mass Index (kg/m^2^). BMD will be measured at the LS (L1-L4) and FN. Additional instructions for DXA imaging will be standardized in a site-specific SOP.

### Extended Follow-Up Subset Procedures

At select sites, consenting participants will be scheduled to return for two 5-week intervals at 10 and 12 months from the last study injection to monitor for return to ovulation. The first 5-week interval will be scheduled to occur 38 weeks from the date of the last injection (i.e., month 8 re-injection date + 38 weeks) with subsequent weekly visits occurring up to 42 weeks or return to ovulation, whichever comes first. If ovulation does not return within the 42 weeks from last injection, participants will be scheduled to return for a second 5-week interval scheduled to occur 46 weeks from the date of their last injection (i.e., month 8 re-injection date + 46 weeks) with subsequent weekly visits occurring up to 50 weeks (12 months from last injection). At predetermined time points (specified in Table 3), blood will be collected for progesterone, estradiol, MPA and other synthetic steroids (e.g., levonorgestrel, etonogestrel and ethinyl estradiol); urine pregnancy testing will be performed; and blood pressure and weight will be measured. Participants will be discontinued when ovulation is confirmed (i.e., defined as a single elevated serum progesterone [P ≥4.7 ng/mL] or confirmed pregnancy test) or at 50 weeks from last injection, whichever comes first.

### Missed and Unscheduled Visits

Re-injection and final visits will be scheduled to occur based on specific days since treatment initiation, allowing for up to a plus 7-day grace period. If a participant cannot return to the clinic within this visit window, the study staff will attempt to have the participant return as soon as possible. However, treatment will be stopped and the participant discontinued from the study if she is more than 28 days late for a re-injection visit. Sites will also ask participants to return to clinic for unscheduled visits if they suspect that they may be pregnant or if they have any other problems or concerns with study participation.

### Post-Discontinuation Visits

Post-discontinuation visits may be conducted if necessary to ensure or monitor participant safety or to assess the EDF of women who report being pregnant within 3 months of having exited the study.

### Loss to Follow Up

If a participant does not appear for a scheduled visit, at least three attempts to contact her will be made. If she does not return to clinic while the study is ongoing then she will be considered potentially lost to follow up, but her file will remain open until study closeout. If the participant does not return before the study is closed, she will be classified as lost to follow up. The lost to follow up designation cannot be made for any participant until the closing date of the study.

# SELECTION AND WITHDRAWAL OF PARTICIPANTS

The target population is sexually active women between 18 and 35 years of age who are willing to use Sayana® Press injected every 4 months as their only means of contraception for 12 months.

## Inclusion Criteria

Women may be included in the study if they meet all of the following criteria at enrollment:

- - not pregnant and no desire to become pregnant in the next 18 months
  - regular menstrual cycles (25 to 35 days in length when not using hormonal contraception, pregnant, or lactating)
  - at risk of pregnancy (no diagnosis of infertility, no history of tubal ligation or hysterectomy, and an average of 1 or more unprotected acts of vaginal intercourse per month)
  - in good general health as determined by a medical history
  - 18 to 35 years of age, inclusive
  - willing to provide informed consent, follow all study requirements, and rely on Sayana® Press injected every 4 months as her sole means of contraception for 12 months
  - has only one sexual partner and expects to have that same sexual partner for the next 12 months

## Exclusion Criteria

Women will be excluded from this study if they meet any of the following criteria at enrollment:

- has a primary partner who has received a vasectomy or is otherwise sterile
- medical contraindications to depot medroxyprogesterone acetate (DMPA) per WHO medical eligibility criteria for contraceptive use [[33](#_ENREF_33)]
- known HIV-infection (for her or a partner)
- diagnosis or treatment for an STI in the past month (for her or a partner), excluding recurrent herpes or condyloma
- received an injection of a progestin-only containing contraceptive (DMPA or norethisterone enanthate) in the past 12 months
- received an injection of a combined injectable contraceptive in the past 6 months
- known or suspected allergic reaction to DMPA
- used a levonorgestrel intrauterine system, NuvaRing, contraceptive patch, oral contraceptives or a contraceptive implant in the past 7 days (PK cohort only)
- Previous (within 1 month prior to enrollment), current or planned (in the next 12 months) use of an investigational drug, prohibited drug per protocol (see Section 5.3) or other drug which in the opinion of the investigator could complicate study findings
- has been pregnant in the past month
- is lactating
- plans to move to another location in the next 12 months
- has a social or medical condition which in the opinion of the investigator would make study participation unsafe, or interfere with adherence to protocol requirements.

## Justification for Key Inclusion and Exclusion Criteria

Only women reasonably expected to be at risk of pregnancy in the absence of using the study product will be enrolled so as to estimate the contraceptive efficacy of the regimen. Women at high risk of HIV and/or STIs will be excluded to limit condom use during the treatment period. Since DMPA is associated with an extended delay in return to fertility, women who have used DMPA-IM or Depo-SubQ Provera 104® in the previous 12 months will be excluded. The study will also not enroll women who have a medical or social condition that may make study participation unsafe or complicate data interpretation according to the site investigator’s assessment.

## Withdrawal Criteria and Procedures

Participants may withdraw from study participation at any time without loss of other benefits or services to which they would otherwise receive as part of standard clinical care. The site investigator can also withdraw a participant from the study for the safety of the participant. Participants who return to the clinic more than 28 days late for a re-injection visit will be discontinued from the study. Participants will also be withdrawn if the study is terminated early. Reasons for withdrawal from the study will be recorded on CRFs. When early discontinuation from the study occurs, the final visit procedures will be followed and every reasonable effort will be made to assess information relevant to the primary and secondary endpoints at the time of discontinuation. Post-discontinuation visits may be conducted if necessary to ensure participant safety or to assess the EDF of women who report being pregnant within 3 months of having exited the study

Participants who withdraw early will not be replaced. However, consideration will be given to expanding enrollment if deemed necessary to ensure that the study objectives regarding the number of evaluable participants completing 12 months of treatment and precision of the Pearl Index are achieved.

# TREATMENT OF PARTICIPANTS

## Study Drugs Administered During the Study

Each participant will receive an injection of Sayana® Press at enrollment, month 4, and month 8, with SC injection site determined by randomization. Instructional materials developed by PATH, which are consistent with prescribing information, will be followed when providing injections [[32](#_ENREF_32)]; Appendix 1).

## Restrictions

Medications prohibited before and during the study are described in Section 5.3. Participants will remain at the study clinic for at least 15 minutes after study drug injection for observation for possible allergic reaction and/or injection site reaction. The study site will have an EpiPen® (or generic epinephrine injection) available and access to emergency care in the unlikely event of an anaphylaxis or anaphylactoid reaction.

## Prior and Concomitant Therapy or Medication

Study staff must record on the corresponding CRF use of any study prohibited concomitant medications and other contraceptives the participant takes during the study. Indication, dosage, and start and end dates will be entered on the corresponding CRF. Use of the following medications is an exclusion criterion at enrollment, and participants should not initiate their use during study participation unless prescribed by a physician:

- Rifampicin
- Griseofulvin
- Anticonvulsants
- Barbiturates
- Non-nucleoside reverse transcriptase inhibitors (efavirenz, nevirapine) and ritonavir-boosted protease inhibitors
- Aminoglutethimide
- St. John’s wort
- Steroids (synthetic estrogens, progestins, androgens)
- Use of selective COX-2 inhibitors (e.g., meloxicam, celecoxib) for 5 or more consecutive days

## Procedures for Monitoring Participant Treatment Compliance

Participant compliance with treatment will be monitored by tracking attendance at scheduled re-injection visits and documenting the dates and anatomical site of re-injection.

# ASSESSMENT OF EFFICACY

Contraceptive efficacy of Sayana® Press administered at 4-month intervals will primarily be evaluated based on the pregnancy Pearl Index over the 12-month treatment period. Any pregnancy outcome which does not undergo confirmatory testing, including self-reports, will be included in the primary assessment of efficacy unless the site investigator and study clinician unambiguously determine that the diagnosis was incorrect or that self-report was not reliable. Women who suspect pregnancy in the first 3 months after study participation has ended will be asked to return to the clinic to determine if fertilization may have occurred while still in the study; any such pregnancies with EDF that fall in the treatment period (as defined in Section 10.6) will be included in the primary efficacy assessment.

# ASSESSMENT OF PHARMACOKINETICS

## Pharmacokinetic Measures

The PK of MPA when Sayana® Press is injected every 4 months will be evaluated based on serum MPA concentrations collected at baseline (day 0) and months 2, 3, 4, 8, and 12 among the subset of N=120 women in the PK cohort. PK measures, including 4-month trough concentrations, accumulation ratio, and apparent half-life will be summarized by SC injection site. Any participant with more than 0.05 ng/mL MPA in her baseline specimen will be excluded from the primary PK analysis.

## Blood Sampling, Handling, and Laboratory Analysis

Blood samples from all participants at enrollment, participants in the PK cohort, and participants who become pregnant, will be collected via venipuncture for serum MPA concentration measurements. The dates and times of each sample will be recorded on appropriate study CRFs and tracking tools. Detailed instructions for preparation of serum samples for MPA testing will be provided in the Study Manual.

One aliquot will remain frozen on site, and another will be sent on dry ice in batches with other samples to PPD Development (Richmond, VA, USA) where MPA concentrations will be measured via a proprietary method utilizing sensitive and selective high-performance liquid chromatography coupled with mass spectrometry. This validated method is applicable to the quantitation of MPA within a nominal range of 0.02 to 5.00 ng/mL, and requires a 250-μL aliquot of human serum.

PPD will maintain source documentation for MPA data. MPA results will be transferred to FHI 360 as password-protected Excel files for analysis. MPA results will not be documented on study CRFs or communicated to the study participants. No further testing of any remaining blood samples is planned; however, any remaining serum will be stored by PPD at least until the end of the study in the event of needed repeat testing or analysis of long-term stored sample stability. The PPD lab will discard remaining blood samples per PPD’s SOPs upon receiving written approval from FHI 360. Aliquots stored on site will also be maintained until the end of the study, and only discarded after receiving written FHI 360 approval.

# ASSESSMENT OF SAFETY

Safety will be assessed by qualified study staff by evaluating the following: serious AEs, AEs leading to product withdrawal, changes in BP, changes in bleeding and changes in body weight.

## Adverse Events

Participants will be provided instructions for contacting the study site to report any untoward medical occurrences they may experience (whether they think it is related or not related to the study product), except for events which they perceive to be emergencies, for which they will be instructed to seek immediate emergency care. Participants will be able to seek evaluation at the study site, where feasible and medically appropriate. The site investigator should provide or refer for medical care, and monitor any clinically significant AE until it has resolved or stabilized. With permission of the participant, and whenever possible, records from all non-study medical providers related to untoward medical occurrences may be obtained by study staff for review.

Any participant who experiences an AE may be withdrawn from study treatment at any time at her will or at the discretion of the site investigator. When early discontinuation from the study occurs, every reasonable effort will be made to assess information relevant to the endpoints at the time of discontinuation and recorded on the Final CRF. Discontinued participants will be monitored at the discretion of the site investigator (e.g., until any clinically significant AE has resolved or stabilized, until the participant is referred to the care of a health care professional, or until a determination of a cause unrelated to the study drug or study procedure is made).

SAEs and AEs resulting in product discontinuation will be recorded on study CRFs and reported to FHI 360. For these AEs the severity will be graded per the Division of AIDS Table for Grading Adult and Pediatric Adverse Events Version 2.0, November 2014. The relationship to the study drug will be characterized as follows:

- *Definitely related*: AEs for which, after careful medical consideration at the time they are evaluated, a connection with the study drug administration is determined with certainty.
- *Possibly related*: AEs for which, after careful medical consideration at the time they are evaluated, a connection with the study drug administration cannot be ruled out with certainty.
- *Not related*: AEs that, after careful consideration, are clearly due to extraneous causes (disease, environment, etc.) or to AEs that, after careful medical consideration at the time they are evaluated, are judged to be unrelated to the study drug.

Departures from the protocol may be allowed on a case by case basis if a participant experiences an AE or medical emergency. After stabilization and/or treatment has been administered to ensure participant safety, the site investigator or other physician in attendance must contact the FHI 360 Study Director as soon as possible to discuss the situation. The site investigator, in consultation with FHI 360, will decide whether the participant should continue to participate in the study despite the protocol deviation. Any departures from the protocol because of AEs will be noted on a Protocol Violation CRF.

## Serious Adverse Events

### Definition of a Serious Adverse Event

An AE is considered an SAE if, in the view of either the site investigator or FHI 360, it results in any of the following outcomes:

- Death
- A life-threatening adverse event (an AE that in the view of the site investigator or FHI 360, places the participant at immediate risk of death)
- Inpatient hospitalization (excluding hospitalization for elective surgery of pre-existing condition) or prolongation of existing hospitalization
- A persistent or significant incapacity or substantial disruption of the ability to conduct normal life functions
- A congenital anomaly/birth defect
- Important medical events that may not result in death, be life-threatening, or require hospitalization may be considered serious when, based upon appropriate medical judgment, they may jeopardize the patient or subject and may require medical or surgical intervention to prevent one of the outcomes listed in this definition

### Reporting SAEs

#### Investigator Responsibilities

SAEs that occur during the study, regardless of judged relationship to treatment with the study drug, must be reported to FHI 360 by the site investigator. All contact information and detailed instructions for SAE reporting will be provided in the Study Manual. The event must be reported within 24 hours of the site learning of the event. Additional information about any SAE unavailable at the initial reporting should be sent to FHI 360 by the site investigator when it becomes known. The SAE will be documented on the appropriate forms, and, in addition to FHI 360, communicated to the local regulatory authorities (if applicable) and local IRBs by the site investigator according to the local requirements.

#### FHI 360 Responsibilities

FHI 360 will report all SAEs in this study to FHI 360’s PHSC per applicable SOPs. If an SAE is believed to be both related to the study drug and unexpected (i.e., not listed in the Prescribing Information for Depo-SubQ Provera 104® or not listed at the specificity or severity that has been previously observed), then FHI 360 will take appropriate steps to notify all site investigators. Details on the SAE reporting will be included in a Safety Management Plan.

## Injection site reactions

The site of new and previous injection(s) will be observed for possible injection site reactions at the enrollment, re-injection and final visits. Following administration of Sayana® Press all participants will stay in the clinic for 15 minutes for observation. All injection site reactions will be graded according to the DAIDS AE severity table. All ISRs that are classified as Grade 1 or higher will be reported as AEs. When feasible and with written consent from the study participant, photographs may be taken of the injection site to supplement documentation of ISRs.

## Return to ovulation (extended follow-up subset only)

The exploratory pharmacodynamics endpoint for this study is ovulation defined as a single elevated serum progesterone (P≥4.7 ng/mL) or a confirmed pregnancy test. Progesterone will be measured weekly during the two 5-weeks intervals for return to ovulation. Pregnancy test will be performed at weeks 38, 42, 46 and 50. Estradiol, an additional pharmacodynamics indicator, will be measured every time progesterone is measured. We will also measure MPA and other hormones (e.g., levonorgestrel, etonogestrel and ethinyl estradiol) at weeks 38 and 46 post last injection to rule out concurrent use of other hormonal contraceptives that may suppress ovarian function. See Table 3 for timing of study procedures.

### Blood Sampling, Handling, and Laboratory Analysis

Blood samples will be collected via venipuncture. The dates and times of each sample will be recorded on appropriate study CRFs and tracking tools. Detailed instructions for preparation of serum samples for MPA testing and plasma for levonorgestrel, etonogestrel and ethinyl estradiol will be provided in the Study Manual.

One aliquot will remain frozen on site, and another will be sent on dry ice in batches with other samples to PPD Development (Richmond, VA, USA) where MPA concentrations will be measured via a proprietary method utilizing sensitive and selective high-performance liquid chromatography coupled with mass spectrometry. This validated method is applicable to the quantitation of MPA within a nominal range of 0.02 to 5.00 ng/mL, and requires a 250-μL aliquot of human serum. Levonorgestrel, etonogestrel and ethinyl estradiol will be measured as well.

PPD will maintain source documentation for all data. Results will be transferred to FHI 360 as password-protected Excel files for analysis. Results will not be documented on study CRFs or communicated to the study participants. No further testing of any remaining blood samples is planned; however, any remaining serum will be stored by PPD at least until the end of the study in the event of needed repeat testing or analysis of long-term stored sample stability. The PPD lab will discard remaining blood samples per PPD’s SOPs upon receiving written approval from FHI 360. Aliquots stored on site will also be maintained until the end of the study, and only discarded after receiving written FHI 360 approval.

## Pregnancy outcomes

A urine pregnancy test will be performed at enrollment, month 4, the final visit, if a woman is more than 7 days late for her month 8 visit, or if she is experiencing any symptoms or signs of pregnancy. A blood specimen for MPA testing will be collected at any time of a clinic-based positive pregnancy test during follow-up. Any participant who becomes pregnant during the study will be discontinued from regular follow-up and pregnancy recorded as reason for study completion on appropriate study CRFs. Participants who become pregnant will be monitored to the completion or termination of the pregnancy or longer if required by local regulations or IRBs. Participants who become pregnant will be asked to contact the study site at the time of pregnancy resolution to provide information on the pregnancy outcome including spontaneous or voluntary termination, details of birth and presence or absence of any birth defect, congenital abnormalities, or maternal and newborn complications. Approximately one month after the estimated date of delivery, women who have not contacted the study site will be contacted to attempt collection of pregnancy outcome information.

## Bleeding Assessment

Bleeding patterns will be assessed and recorded on the appropriate CRF at baseline, months 4, 8, and final visit.

## Measurement of Body weight and BP

Measurement of body weight and BP, will be performed at baseline and at regularly scheduled visits (months 4, 8, and final) (See Table 2), and documented on appropriate CRFs. Measurement of body weight and BP will also be performed at weeks 38 and 42 (extended follow-up subset only [see Table 3]).

## Concomitant Medication

Use of study prohibited concomitant medication and other contraceptives will be monitored and recorded on appropriate study CRFs throughout the study. Details of prohibited medications are found in Section 5.3.

## Social Harm Events

A social harm event is an adverse social consequence or outcome due to study participation. If a site investigator learns of a social harm event that is serious, unexpected and judged to be related or possibly related to the study protocol he/she will report the event to FHI 360 within 24 hours of becoming aware of the event. FHI 360 will report the event to FHI 360’s PHSC.

## Methods and Timing of Assessing, Recording, and Analyzing Safety Data

Methods and timing of assessing safety data are discussed in Section 3.14. Procedures for recording safety data are discussed in Section 8, above, and methods of analysis are discussed in Section 10.

# ASSESSMENT OF ACCEPTABILITY

The acceptability of a Sayana® Press injected every 4 months will be assessed and recorded on appropriate study CRFs at regularly scheduled visits (months 4, 8, and final). Acceptability questions will include questions such as acceptability of bleeding pattern and whether the participant would use the method in the future.

# STATISTICS

Details of the planned statistical analyses to address the study objectives will be provided in a separate statistical analysis plan (SAP) developed prior to implementation of the trial. The following is a summary of the planned analyses of the study.

## Sample Size and Power Considerations

The sample size of 710 women receiving injections in the abdomen or thigh is expected to be sufficient to ensure that the difference between the estimated Pearl Index and the corresponding upper 95% confidence bound does not exceed 1.0, so long as the observed index is less than 0.75 and at least 80% of participants complete 12 months of follow-up. In addition, a cohort of 120 women (40 per SC injection site) is sufficient to provide 85% power to detect 30% relative differences in PK parameters between injection sites, assuming the corresponding coefficient of variation is no more than 40% and using two-sided 0.05 significance tests.

## Analysis Populations and Analysis Sets

In the below definitions, an Analysis Population refers to a particular set of participants and an Analysis Set defines the time contributed to analyses by participants in a given Population. Additional censoring rules for analyses based on these sets are described in relevant sections of the SAP.

### Treated Population and Analysis Set

The Treated Population consists of all screened participants who are enrolled and receive a dose of study drug. For the primary efficacy analysis, the corresponding Treated Analysis Set includes all follow up time contributed by each participant in the Treated Population until their EDF, the end of their treatment period, or the date of their last negative urine pregnancy test (for participants who do not become pregnant), whichever is earlier. Comparative efficacy analyses based on SC injection site will be performed according to intention-to-treat principles, ignoring any randomization or allocation errors.

### Pharmacokinetics Population and Analysis Set

The Pharmacokinetics Population is a subset of the Treated Population, excluding participants who did not contribute blood specimens for PK assessments or who had a baseline serum MPA concentration exceeding 0.05 ng/mL. The corresponding Pharmacokinetics Analysis Set will censor data collected after a participant receives an injection in an anatomical site which differs from the site used at enrollment and will exclude any MPA specimens collected during a time when a participant reports use of a concomitant medication that may impact the PK of MPA. PK analyses based on this analysis populationconmed will be performed according to the SC site of injection at enrollment, even if received in error.

### Extended Follow-up Population

The Extended Follow-up Population is a subset of the Treated Population, including participants who meet criteria for the extended follow-up subset and consent to remain in follow-up past their month 12 visit in order to provide information on return to ovulation.

## Data Handling Conventions

Estimated dates of fertilization will be adjudicated following FHI 360 SOPs based on ultrasound reports, LMP, or best clinical judgment (if ultrasound results are not available). It is anticipated that some event dates required for safety analyses may be incomplete. For example, participants may not recall the exact day of onset for a self-reported AE. Decision rules for how and when to impute such incomplete date fields will be developed by the medical monitor when reviewing study data and be documented in the study report. Other missing outcome or predictor variables will be ignored unless patterns are identified which suggest the potential for biased assessments of efficacy, safety, PK or acceptability. In that event, rules for imputing data will be clearly described and justified in the study report.

## Multiple Comparisons and Multiplicity

There is a single primary assessment in this study: the pregnancy Pearl Index among women randomized to receive injections in the abdomen or thigh. All confidence intervals reported for the primary, secondary, and subgroup analyses will be computed at the 95% coverage level, and all p-values will be assessed at the two-sided 0.05 significance level, with no adjustment for multiple testing.

## Assessment of Study Populations

Participant disposition, demographic, and other baseline characteristics of the Treated and Pharmacokinetics Analysis Populations will be provided. Summaries will be presented by SC injection site, injection site within study clinic, and pooled across injection sites and study clinics.

### Participant Disposition

The numbers of participants screened, participants screened but not enrolled (including reason(s) not enrolled), participants who withdraw early (and reason), and participants who complete the study will be summarized using descriptive statistics.

### Demographic and other Baseline Characteristics

Demographic and baseline characteristics, including medical history and concomitant medication use, will be summarized using frequencies and percentages for categorical data and means, standard deviations, standard errors, medians, minima, and maxima for continuous variables. Differences in distributions of these characteristics between clinic sites will be assessed using exploratory analysis of variance for continuous variables and Chi-squared or Fisher’s Exact tests for categorical variables.

## Efficacy Analysis

### Primary Endpoint

Pregnancies will be detected by urine pregnancy tests conducted at site visits and confirmed by ultrasound and/or serum hCG testing. The EDF for each pregnancy will be calculated by a qualified study clinician based on ultrasound and LMP. In the absence of ultrasound or LMP, best clinical judgment will be used to estimate the EDF based on all available related information including but not limited to vaginal bleeding, patterns of coitus, and the timing of previous negative pregnancy test results. Time to pregnancy will be computed as the number of days between study enrollment and EDF. Recognizing the plus 7-day grace period for re-injections, pregnancies with an EDF up to 18 weeks (126 days) after the first (enrollment) injection or up to 19 weeks (133 days) after subsequent re-injections will be included in the primary efficacy analysis.

Information on any pregnancies, miscarriages or abortions diagnosed outside the study clinic will be collected during follow-up interviews. Any such pregnancies with EDF determined to be within the treatment period defined above will be included in the primary efficacy analysis unless the site investigator and study clinician unambiguously determine that the pregnancy diagnosis was incorrect and/or self-reported data was unreliable.

### Planned Methods of Analysis

#### Primary Efficacy Analysis

The primary efficacy analysis will be based on the pregnancy Pearl Index, computed as the number of pregnancies that occur during the treatment period multiplied by 100 and divided by the number of WY of treatment contributed to the Treated Analysis Set among women receiving injections in the abdomen or thigh. Participants who do not become pregnant or who have EDFs that fall outside the windows used to define primary outcomes will be censored on the date of last negative urine pregnancy test or the last day of the treatment period of last injection (126 days after first injection or 133 days after injections at months 4 or 8), whichever is earlier. An exact 95% confidence interval for the Pearl Index will be computed based on a Poisson distribution assumption.

#### Secondary Efficacy Analyses

Secondary efficacy analyses will include:

- Typical use Life-Table estimate of the cumulative probability of pregnancy through 3 treatment cycles in the Treated Population (excluding women randomized to the upper arm)
- Pearl Index that excludes follow-up periods during which participants report using other contraceptives (including condoms), drugs known to impact ovarian function, or drugs known to impact the PK of MPA, unless she became pregnant in that period
- Pearl Index that includes any pregnancies with EDF that fall in an 8-28 day ‘late’ period for reinjections or final visits, as well as all person-time (regardless of pregnancy status) contributed to these periods
- Pearl Index computed per the primary analysis, but including women randomized to receive injections in the upper arm

In addition to the non-comparative analyses listed above, a Pearl Index will be computed separately by anatomical site of injection (abdomen or thigh), and the difference in pregnancy risk between site of injection will be assessed using a log-rank test, stratified on clinic site.

## Pharmacokinetic Analysis

### Pharmacokinetic Endpoints

Pharmacokinetic endpoints include serum MPA concentrations at month 2, 3, 4, 8, and 12.

### Planned Method of Analysis

Serum MPA concentration data falling within defined sampling windows will be summarized graphically using box plots, by SC injection site and overall. Trough concentrations at months 4, 8, and 12 will also be summarized using geometric means, standard deviations, and percent coefficient of variation. Month 2, 3, and 4 data will be used to estimate t_1/2_ based on random effects log-linear models (under the assumption that the terminal phase of absorption begins by month 2), including random intercept and slope terms. Accumulation of MPA concentrations at months 8 and 12 will be estimated according to the ratio of trough concentrations and using the formula R=[1-(0.5) ^dosing interval/half-life^]^-1^, where the dosing interval is 126 days. Anatomical sites of injection will be compared by computing 95% confidence intervals for geometric mean ratios of t_1/2_ and trough concentrations. The effects of site, body mass index, and age on PK of MPA will be explored graphically and using analysis of covariance.

## Safety Endpoints and Analysis

### Safety Endpoints

The overall safety of study drug will be assessed throughout the study by evaluating the following endpoints and variables in the Treated Analysis Set:

- occurrence of SAEs and AEs leading to product discontinuation
- occurrence of ISRs
- BP and weight at regularly scheduled visits
- changes in bleeding pattern

### Safety Analysis

All SAEs, AEs leading to product withdrawal, and AEs that are Grade 1 or higher ISRs will be coded by a trained medical coder at FHI 360 using the Medical Dictionary for Regulatory Activities (MedDRA) by preferred term and system organ class. The numbers and percentages of participants experiencing AEs leading to product withdrawal and AEs that are Grade 1 or higher ISRs will be presented by severity. All relevant information on any deaths or other SAEs will be discussed in the subject narrative included in the clinical study report. Numbers and percentages of participants experiencing any ISR will be summarized by SC injection site, and ISRs will also be presented in a listing. Body weight, BP, and bleeding patterns will be summarized graphically or using shift-tables (i.e., change from baseline).

## Acceptability Variables and Analysis

Perception of bleeding patterns and other responses to acceptability questions will be summarized using descriptive statistics based on routine questions asked at regularly scheduled visits (enrollment, months 4 and 8, and final).

## Exploratory Variables and Analysis

To evaluate the exploratory objective of return to ovulation, the proportion of participants in the Extended Follow-up Population who have returned to ovulation will be summarized at 10 months (42 weeks) and 12 months (50 weeks) after final injection.

## BC and BMD Variables and Analysis

Brazil site investigators will develop an analysis plan for the BC-BMD Study. FHI 360 will review and approve the analysis plan prior to data transfer to the site.

## Planned Interim Analysis

An independent DSMB will review interim data when 250 participants have completed 4 months of follow-up. This single, pre-planned interim analysis is intended to inform a decision to stop or modify the study if the estimated probability of pregnancy is greater than 2% in the first injection interval. Consideration may also be given to expanding enrollment if it is deemed necessary to ensure that study objectives regarding the number of evaluable participants completing 12 months of treatment and the desired precision of the Pearl Index are achieved. The interim 4-month PK data, including serum MPA concentrations of any women who become pregnant, may be reviewed by FHI 360 (after the DSMB has deliberated) to inform other FHI 360 MPA projects.

Additional interim analyses may be requested by FHI 360 or the DSMB based on periodic reviews of study indicators (pregnancy, AE, recruitment and continuation rates) if deemed necessary to protect participant safety or to make recommendations to modify the study. Further details of interim monitoring procedures will be provided in a separate DSMB Operational Plan.

## Reporting Deviations from the Statistical Plan

Deviations from analyses described here, along with their reasons, will be provided in protocol amendments, the separate SAP, the clinical study report, or a combination of these, as appropriate.

# DIRECT ACCESS TO SOURCE DATA/DOCUMENTS

Study monitors, auditors, IRBs, and health authority inspectors will be given direct access to source data and documentation (e.g., medical records, laboratory test results) for source data verification, provided that participant confidentiality is maintained in accordance with local requirements.

The site investigator must maintain the original records (i.e., source documents) of each participants’ data at all times. Examples of source documents are signed informed consent forms, clinical notes, laboratory reports and CRFs that are used as the source. Source documents for each participant and original CRFs will be retained by the site investigator per guidelines specified in Section 14.3 of this protocol. No records may be destroyed without written permission from FHI 360.

# QUALITY CONTROL AND QUALITY ASSURANCE

## Protocol Amendments

No changes from the final approved protocol will be initiated without the prior written approval by the governing IRBs, except when necessary to address immediate safety concerns to the participants or when the change involves only logistics or administration. The site investigator will sign the protocol amendment investigator signature page prior to implementation.

## Protocol Violations

Protocol violations which may significantly impact the accuracy and/or reliability of study data or that may significantly affect a participant's rights, safety, or well‑being will be recorded on study CRFs and reported to IRBs and regulatory authorities per requirements.

Emergency departures from protocol that eliminate an apparent immediate hazard to participants and are deemed crucial for the safety and well-being of a participant may be instituted for that participant only by the site investigator. In those cases, the site investigator will notify the local IRB and FHI 360 in writing as soon as possible and document the reasons for the violation.

## Information to Study Personnel

The site investigator is responsible for giving information about the study to all staff members involved in the implementation of the study or in any element of participant management, both before starting the study and during the course of the study (e.g., when new staff become involved). The site investigator must assure that all study staff members are qualified by education, experience, and training to perform their specific responsibilities. All study staff will have training in research ethics and Good Clinical Practice. These study staff members must be listed on the staff responsibility log, which includes a clear description of each staff member’s responsibilities. FHI 360 is responsible for explaining the protocol to all study staff, including the site investigator, and for ensuring they comply with the protocol.

## Study Monitoring

Qualified clinical monitors will conduct periodic study monitoring in accordance with FHI 360 policies. Details of timing and duration of monitoring visits will be described in a clinical monitoring plan. Briefly, clinical monitors will:

- Review informed consent forms and documentation
- Assess compliance with the study protocol, GCP, and applicable regulatory requirements
- Verify that all SAEs have been recorded and reported properly and in accordance with applicable regulatory guidelines
- Perform source document verification to ensure the accuracy and completeness of study data
- Verify proper storage of biological specimens
- Verify proper storage, dispensing, and accountability of study products
- Verify that current credentials are available on site for study staff listed on the current Statement of Investigator Form and Delegation of Responsibilities Log

Site investigators and study staff will allow the clinical monitors to inspect study facilities and documentation including the regulatory file, consent forms, CRFs, and other source documents for verification of the study data. The site investigator and assisting staff must agree to cooperate with the study monitor to resolve any problems, errors, or possible misunderstandings concerning the findings detected in the course of these monitoring visits and/or provided in follow up written communication. As part of the supervision of study progress, other FHI 360 staff or representatives may accompany the study monitor on visits to the study site. Sites will maintain a site visit log to document all visits.

## Audit and Inspection

FHI 360 or regulatory authorities may audit the study sites to evaluate study conduct and compliance with the protocol, SOPs, GCP, and applicable IRB and regulatory requirements.

# ETHICS

## Informed Consent

A participant will not be enrolled into this research study until the site investigator has obtained her signed informed consent (including consent to additional visits if in the PK cohort). The site investigator shall seek such consent under circumstances that provide the prospective participant with sufficient opportunity to learn about the study and consider whether or not to participate. Informed consent will be obtained without coercion, undue influence or misrepresentation of the potential benefits and risks that might be associated with participation in the research study. Informed consent encompasses all oral or written information given to the participant about the study and the study materials. This includes the consent form(s) signed by the participant and any other information provided to the participant. All such information that is given to the participant will be in a language that is understandable to her. The participant must agree that she understands the investigational nature of the study, its inherent risks and benefits, other treatment alternatives, her rights to terminate participation in the study without affecting her health care at the site, whom to contact with questions regarding the study, and that she has freely given informed consent to participate in the study and to have her medical records reviewed as part of the study.

Informed consent will be documented by the use of a written consent form (or forms) signed by the participant. Copies of signed informed consent forms will be given to her. The original signed consent form(s) for each participant will be stored in a secure location separately from the study data forms. The consent form(s) must include each of the basic and additional elements of informed consent described in 45 CFR Part 46.116. The informed consent form will include a statement that personal identifying information will be removed from participant data and the non-identified study data will be shared with the donor, USAID, and might be used for future research. The information will not include any language in which the participant is made to waive any of her rights or which releases or appears to release the site investigator, the site investigator’s institution, or FHI 360 from liability for negligence.

## Compensation

Participants will be compensated for time and effort for participation in this study. Reimbursement will cover transportation costs and time away from work, and may vary by site depending on local ethical review requirements. Reimbursement amounts will be specified in the site-specific informed consent forms and reviewed with participants upon study enrollment.

## Health Authorities and IRBs

This protocol, the informed consent forms, and other appropriate documentation will be reviewed and approved as required by the PHSC at FHI 360 and the site IRBs. The site investigator is responsible for ensuring that all requirements of the local IRB are met. Before implementing any changes to the protocol, informed consent, or participant written materials, the site investigator must have the changes approved by FHI 360’s PHSC and the local IRB, and applicable health regulatory agencies, except where necessary to eliminate immediate hazards to study participants. If the local IRB withdraws its approval of this research at any time before its completion, the site investigator must notify FHI 360 as soon possible, but no later than within 48 hours.

The site investigator is responsible for providing progress reports to the local IRB and to FHI 360 annually and within 3 months after study termination or completion. The reports should include at a minimum the total number of participants enrolled, the numbers and reason(s) for discontinuation, a description of all SAEs, the number of participants completing the study, all changes in the research activity, and all unanticipated problems involving risks to study participants or others. Copies of all study-related correspondence with the local IRB must be sent to FHI 360.

## Study Participant Confidentiality

The site investigator must assure that the privacy of the participants, including their identity and confidentiality of all personal medical information, will be maintained at all times. In CRFs and other documents or image material submitted to FHI 360, participants will be identified not by their names, but by a participant ID number only.

Personal medical information may be reviewed for the purpose of participant safety and/or verifying data in the source and transcribed onto the CRF. This review may be conducted by the study monitor, properly authorized persons on behalf of the sponsor, the quality assurance unit, and/or regulatory authorities. Personal medical information will always be treated as private and confidentiality will be maintained.

## Registration of the Clinical Study

FHI 360 will register this trial on clinicaltrials.gov, a registry and results database of clinical studies of human participants conducted globally.

# DATA HANDLING, DATA QUALITY CONTROL, AND RECORD KEEPING

A detailed data management plan will be written prior to study initiation. The following is a brief summary of the plan.

## Data Collection

Data will be collected using CRFs that are specifically designed for this study. The data collected on the CRFs will be captured in a clinical data management system (CDMS) that meets the technical requirements described in 21CFR Part 11. The CDMS has been validated to ensure that it meets the scientific, regulatory, and logistical requirements of the study before it is used to capture data from this study. Before using the CDMS, all users will receive training on the system and study specific training. After they are trained, users will be provided with individual system access rights.

Data will be collected at the study site by appropriately designated and trained personnel, and CRFs must be completed for each screened participant. For participants who enter the study but do not meet screening criteria, at a minimum, data for screen failure reason will be entered onto a CRF.

Participant identity should not be discernible from the data provided on the CRFs. Data will be verified by the monitor and reviewed for consistency by Data Management using both automated logical checks and manual review. All data collected will be approved by the site investigator or designee at the study site. This approval acknowledges the review and acceptance of the data as being complete and accurate.

Data processed from PPD will be sent electronically to FHI 360 for direct import into the clinical database, but not entered onto the CRF unless otherwise specified in the protocol.

## Data Quality Control

Data Management is responsible for the accuracy, quality, completeness, and internal consistency of the data from this study. Data handling, including data quality control, will comply with international regulatory guidelines, including ICH GCP guidelines. Data management and control processes specific to this study, along with all steps and actions taken regarding data management and data quality control, will be described in a data management plan.

Case report forms received will be processed and reviewed for completeness, consistency, and the presence of mandatory values. Applicable terms will be coded according to the coding conventions for this study. Logical checks will be implemented to ensure data quality and accuracy. Any necessary data changes will be made in the clinical database, and data review and validation procedures will be repeated as needed. Discrepancies found will be queried.

Data corrections will be made using the CDMS Discrepancy module. The system audit trail captures the reason for each change, the previous and new data values, date and time, and the username of the staff person making the change.

At the conclusion of the study, the CDMS and all other study data will be locked to prevent further changes. Locking the study data represents the acknowledgement that all data have been captured and confirmed as accurate.

## Archiving of Case Report Forms and Source Documents

### Investigator Responsibilities

All records related to the study (i.e., source data, source documents, CRFs, copies of protocols and protocol amendments, drug accountability forms, correspondence, signed informed consent forms, and other essential documents) must be retained by the site investigator. Per GCP, essential documents should be retained until at least 2 years after the last approval of a marketing application in an ICH region and until there are no pending or contemplated marketing applications in an ICH region or at least 2 years have elapsed since the formal discontinuation of clinical development of the investigational product. FHI 360 will inform the site investigator as to when these documents no longer need to be retained. No records may be destroyed without written permission from FHI 360. Should the site investigator wish to assign the study records to another party or move them to another location, advance written notice will be given to FHI 360.

### FHI 360 Responsibilities

FHI 360 will be responsible for the processing and quality control of the data. Data management will be carried out as described in FHI 360 SOPs and the data management plan.

# FINANCING AND INSURANCE

A separate financial agreement will be made between FHI 360 and the clinical sites before the study drug is delivered.

The study will comply with requirements of US and the countries where the study is conducted regarding liability insurance coverage, as applicable.

# **REPORTING AND PUBLICATION OF RESULTS**

There will be a primary publication of the study led by FHI 360 reporting the results of the primary and secondary objectives stated in the study protocol, which must precede any secondary publications or presentations of the results by the participating site investigators. Collaborators will discuss and agree upon any planned publication before submission. The study sites may not publish data collected in this study, or any sub-studies, without prior written approval from FHI 360.

All publications, reports and official presentations will include the following statement "This (study/report/visual/other information/media product) is made possible by the generous support of the American people through the U.S. Agency for International Development (USAID). The contents are the responsibility of FHI 360 and do not necessarily reflect the views of USAID or the United States Government.”

Authorship roles will be assigned primarily according to the contributions of individual members. Other key contributors to the research will be acknowledged as part of the research team. Results will be presented at relevant conferences and published in peer-reviewed journals as open access articles unless a waiver is granted by USAID.

# REFERENCES

1. Stanback, J., et al., *Community-based health workers can safely and effectively administer injectable contraceptives: conclusions from a technical consultation.* Contraception, 2010. **81**(3): p. 181-184.

2. Hatcher, R.A., et al., *Contraceptive Efficacy*. Eighteenth Revised Edition ed. Contraceptive Technology. 2004, New York, NY: Ardent Media.

3. World Health Organization (WHO), *Selected Practice Recommendations for Contraceptive Use, 2008 Update*. 2008: Geneva.

4. Kaunitz, A.M., *Long-acting injectable contraception with depot medroxyprogesterone acetate.* Am J Obstet Gynecol, 1994. **170**(5 Pt 2): p. 1543-9.

5. Kaunitz, A.M., *Long-acting hormonal contraception: assessing impact on bone density, weight, and mood.* Int J Fertil Womens Med, 1999. **44**(2): p. 110-7.

6. Xiang, A.H., et al., *Long-acting injectable progestin contraception and risk of type 2 diabetes in Latino women with prior gestational diabetes mellitus.* Diabetes Care, 2006. **29**(3): p. 613-7.

7. Pfizer, Inc., *Depo Provera CI (medroxyprogesterone acetate) injectable suspension 150 mg/1 mL [prescribing information]*. Revised October 2010, Pharmacia and Upjohn, Division of Pfizer Inc.: New York, NY.

8. Heffron, R., et al., *Use of hormonal contraceptives and risk of HIV-1 transmission: a prospective cohort study.* Lancet Infect Dis, 2012. **12**(1): p. 19-26.

9. Morrison, C.S., et al., *Hormonal contraception and the risk of HIV acquisition: an individual participant data meta-analysis.* PLoS Med, 2015. **12**(1): p. e1001778.

10. Jain, J., et al., *Pharmacokinetics, ovulation suppression and return to ovulation following a lower dose subcutaneous formulation of Depo-Provera.* Contraception, 2004. **70**(1): p. 11-8.

11. Jain, J., et al., *Contraceptive efficacy and safety of DMPA-SC.* Contraception, 2004. **70**(4): p. 269-75.

12. Kaunitz, A.M., et al., *Subcutaneous DMPA vs. intramuscular DMPA: a 2-year randomized study of contraceptive efficacy and bone mineral density.* Contraception, 2009. **80**(1): p. 7-17.

13. Cameron, S.T., A. Glasier, and A. Johnstone, *Pilot study of home self-administration of subcutaneous depo-medroxyprogesterone acetate for contraception.* Contraception, 2012. **85**(5): p. 458-64.

14. Burke, H.M., et al., *Observational study of the acceptability of Sayana(R) Press among intramuscular DMPA users in Uganda and Senegal.* Contraception, 2014. **89**(5): p. 361-7.

15. Shelton, J.D. and V. Halpern, *Subcutaneous DMPA: a better lower dose approach.* Contraception, 2014. **89**(5): p. 341-343.

16. Medicines and Healthcare Products Regulatory Agency (MHRA), *Depo-subQ provera 104® medroxyprogesterone acetate injectable suspension 104 mg/0.65 mL [package insert]*. Revised January 2015 Pharmacia and Upjohn, Division of Pfizer Inc.: New York, NY.

17. Medicines and Healthcare Products Regulatory Agency (MHRA), *Public Assessment Report, Mutual Recognition Procedure, Sayana 104mg/0.65mL suspension for injection*. Revised August 2015.

18. Jordan, A., *Toxicology of depot medroxyprogesterone acetate.* Contraception, 1994. **49**(3): p. 189-201.

19. Toh, Y.C., et al., *Suppression of ovulation by a new subcutaneous depot medroxyprogesterone acetate (104 mg/0.65 mL) contraceptive formulation in Asian women.* Clinical Therapeutics, 2004. **26**(11): p. 1845-1854.

20. Halpern, V., et al., *Pharmacokinetics of subcutaneous depot medroxyprogesterone acetate injected in the upper arm.* Contraception, 2014. **89**(1): p. 31-5.

21. Center for Disease Control and Prevention (CDC), *Update to CDC's U.S. Medical Eligibility Criteria for Contraceptive Use, 2010: revised recommendations for the use of contraceptive methods during the postpartum period*, in *Morbidity and Mortality Weekly Report (MMWR)*. 2011 p. 878-83.

22. Jodicke, A.M., et al., *Severe injection site reactions after subcutaneous administration of Sayana(R).* Swiss Med Wkly, 2017. **147**: p. w14432.

23. Modesto, W., et al., *Weight variation in users of depot-medroxyprogesterone acetate, the levonorgestrel-releasing intrauterine system and a copper intrauterine device for up to ten years of use.* Eur J Contracept Reprod Health Care, 2015. **20**(1): p. 57-63.

24. Polis, C.B., et al., *An updated systematic review of epidemiological evidence on hormonal contraceptive methods and HIV acquisition in women.* AIDS, 2016. **30**(17): p. 2665-2683.

25. World Health Organization (WHO), *Hormonal contraceptive methods for women at high risk of HIV and living with HIV*, in *Guidance Statement*. 2014: Geneva.

26. World Health Organization (WHO), *Hormonal contraceptive methods for women at high risk of HIV* in *Guidance Statement*. 2017: Geneva.

27. Kaunitz, A.M. and D.A. Grimes, *Removing the black box warning for depot medroxyprogesterone acetate.* Contraception, 2011. **84**(3): p. 212-213.

28. World Health Organization (WHO), *WHO statement on hormonal contraception and bone health*, in *Weekly Epidemiological record*. 2005. p. 302-304.

29. Pfizer, Inc., *Sayana® Press Package Leaflet: Information for the User*.

30. Huijbregts, R.P., K.G. Michel, and Z. Hel, *Effect of progestins on immunity: medroxyprogesterone but not norethisterone or levonorgestrel suppresses the function of T cells and pDCs.* Contraception, 2014. **90**(2): p. 123-9.

31. European Medicines Agency (EMA), *Guideline on Clinical Investigation of Steroidal Contraceptives in Women*. 2005: London.

32. PATH, *Sayana Press: A Guide for Trainers of Providers*

33. World Health Organization (WHO), *Medical eligibility criteria for contraceptive use*. 2015: Geneva.

# APPENDICES

## Appendix 1: Sayana**^®^** Press Injection Job Aid


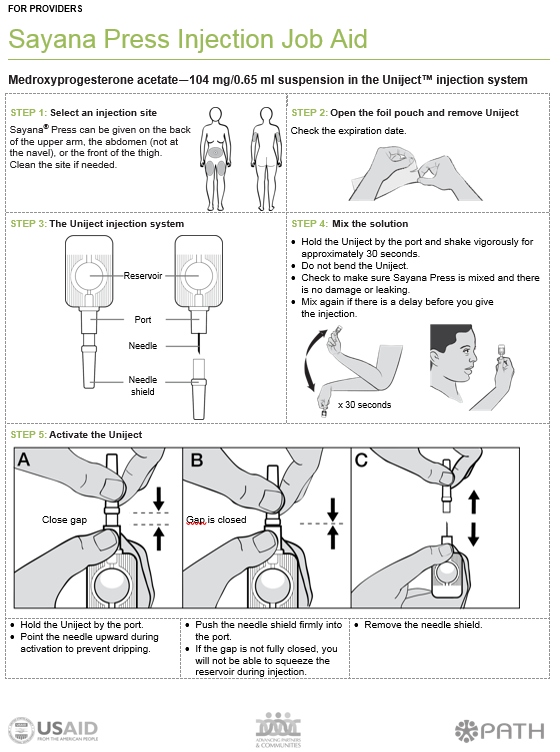


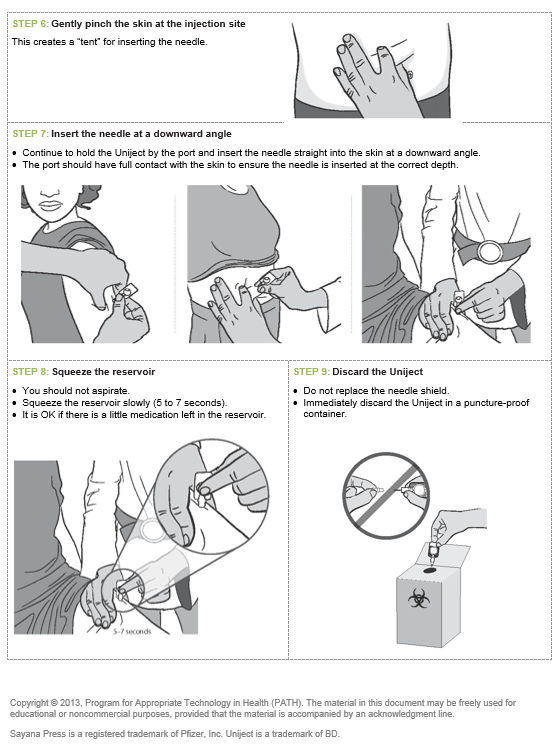

Supplement: Supplementary file 1 [file mmc1.docx]
